# Dinosaur ichnology and sedimentology of the Chignik Formation (Upper Cretaceous), Aniakchak National Monument, southwestern Alaska; Further insights on habitat preferences of high-latitude hadrosaurs

**DOI:** 10.1371/journal.pone.0223471

**Published:** 2019-10-30

**Authors:** Anthony R. Fiorillo, Yoshitsugu Kobayashi, Paul J. McCarthy, Tomonori Tanaka, Ronald S. Tykoski, Yuong-Nam Lee, Ryuji Takasaki, Junki Yoshida

**Affiliations:** 1 Perot Museum of Nature and Science, Dallas, Texas, United States of America; 2 Hokkaido University Museum, Hokkaido University, Hokkaido, Japan; 3 Department of Geosciences, University of Alaska, Fairbanks, Alaska, United States of America; 4 Department of Natural History and Planetary Sciences, Hokkaido University, Hokkaido, Japan; 5 School of Earth and Environmental Sciences, Seoul National University, Seoul, South Korea; Universidade de Sao Paulo, BRAZIL

## Abstract

While there are now numerous records of dinosaurs from Cretaceous rocks around the state of Alaska, very few fossil records of terrestrial vertebrates are known from the Mesozoic rocks of the southwestern part of the state. Here we report the new discovery of extensive occurrences of dinosaur tracks from Aniakchak National Monument of the Alaska Peninsula. These tracks are in the Late Cretaceous (Maastrichtian) Chignik Formation, a cyclic sequence of rocks, approximately 500–600 m thick, representing shallow marine to nearshore marine environments in the lower part and continental alluvial coastal plain environments in the upper part of the section. These rocks are part of the Peninsular Terrane and paleomagnetic reconstructions based on the volcanic rocks of this terrane suggest that the Chignik Formation was deposited at approximately its current latitude which is almost 57° N. Recent field work in Aniakchak National Monument has revealed over 75 new track sites, dramatically increasing the dinosaur record from the Alaska Peninsula. Most of the combined record of tracks can be attributed to hadrosaurs, the plant-eating duck-billed dinosaurs. Tracks range in size from those made by full-grown adults to juveniles. Other tracks can be attributed to armored dinosaurs, meat-eating dinosaurs, and two kinds of fossil birds. The track size of the predatory dinosaur suggests a body approximately 6–7 m long, about the estimated size of the North Slope tyrannosaurid *Nanuqsaurus*. The larger bird tracks resemble *Magnoavipes denaliensis* previously described from Denali National Park, while the smaller bird tracks were made by a bird about the size of a modern Willet. Previous interdisciplinary sedimentologic and paleontologic work in the correlative and well-known dinosaur bonebeds of the Prince Creek Formation 1400km-1500km further north in Alaska suggested that high-latitude hadrosaurs preferred distal coastal plain or lower delta plain habitats. The ichnological record being uncovered in the Chignik Formation of southwestern Alaska is showing that the hadrosaur tracks here were also made in distal coastal and delta plain conditions. This similarity may corroborate the habitat preference model for Cretaceous high-latitude dinosaurs proposed for the data gathered from the Prince Creek Formation, and may indicate that at least Beringian hadrosaurids had similar habitat preferences regardless of latitude.

## Introduction

An inventory and monitoring program initiated by the United States National Park Service, Alaska Region, in 2001 resulted in the first discovery of Cretaceous dinosaurs in southwestern Alaska, as well as the first documentation of any dinosaur record in any of the National Park units in Alaska [1]. This initial discovery prompted additional investigation in other National Park units within Alaska, and there are now numerous documented occurrences of dinosaurs and other fossil vertebrates in Denali National Park in the central Alaska Range [2–12], in Wrangell-St. Elias National Park [13], and Yukon-Charley Rivers National Preserve [14]. While most of these fossil vertebrate occurrences are correlative with the famous fossil bone deposits of the Prince Creek Formation of the North Slope of Alaska [2, 12, 15–18], more recent work has shown that the Wrangell-St. Elias National Park dinosaur record is several 10s of millions of years older [19]. Together these discoveries demonstrate that terrestrial ecosystems capable of supporting dinosaurs occurred across a wide geographic region in the ancient Arctic.

A recent study in the Prince Creek Formation of the North Slope of Alaska integrated detailed examination of depositional processes with the vertebrate body fossil record [20]. That study showed a pattern of dominance of hadrosaur remains in the more distal basin, represented by lower delta plain facies, while ceratopsian remains were more prevalent in the more proximal part of the basin, represented by better drained coastal plain facies. Sediments of the Chignik Formation in Aniakchak National Monument are located approximately 1500 kilometers to the southwest of the Prince Creek Formation sites and are correlative with them. The integration of detailed depositional analysis with dinosaurian-ichnological data in Aniakchak National Monument offers the opportunity to test this model of habitat preference for southern Alaskan dinosaur communities as well.

In this study we document the occurrence of previously unrecognized vertebrate ichnotaxa from the Upper Cretaceous Chignik Formation of southwestern Alaska. Thus this report serves several purposes. First, it expands the known ichnotaxonomic diversity within this rock unit. Second, we expand on previous stratigraphic work in this area and provide new insights into the depositional setting for these vertebrate tracks. Third, these combined data provide an independent test of the model for habitat preference, derived from fossil bone frequencies, for the most commonly occurring northern high-latitude dinosaurs, the hadrosaurs. And lastly, the avian theropod tracks found in this study warrant some discussion regarding ichnotaxonomy and the attribution of the ichnogenus *Magnoavipes*.

## Geographic and geologic background

**Fig 1:**
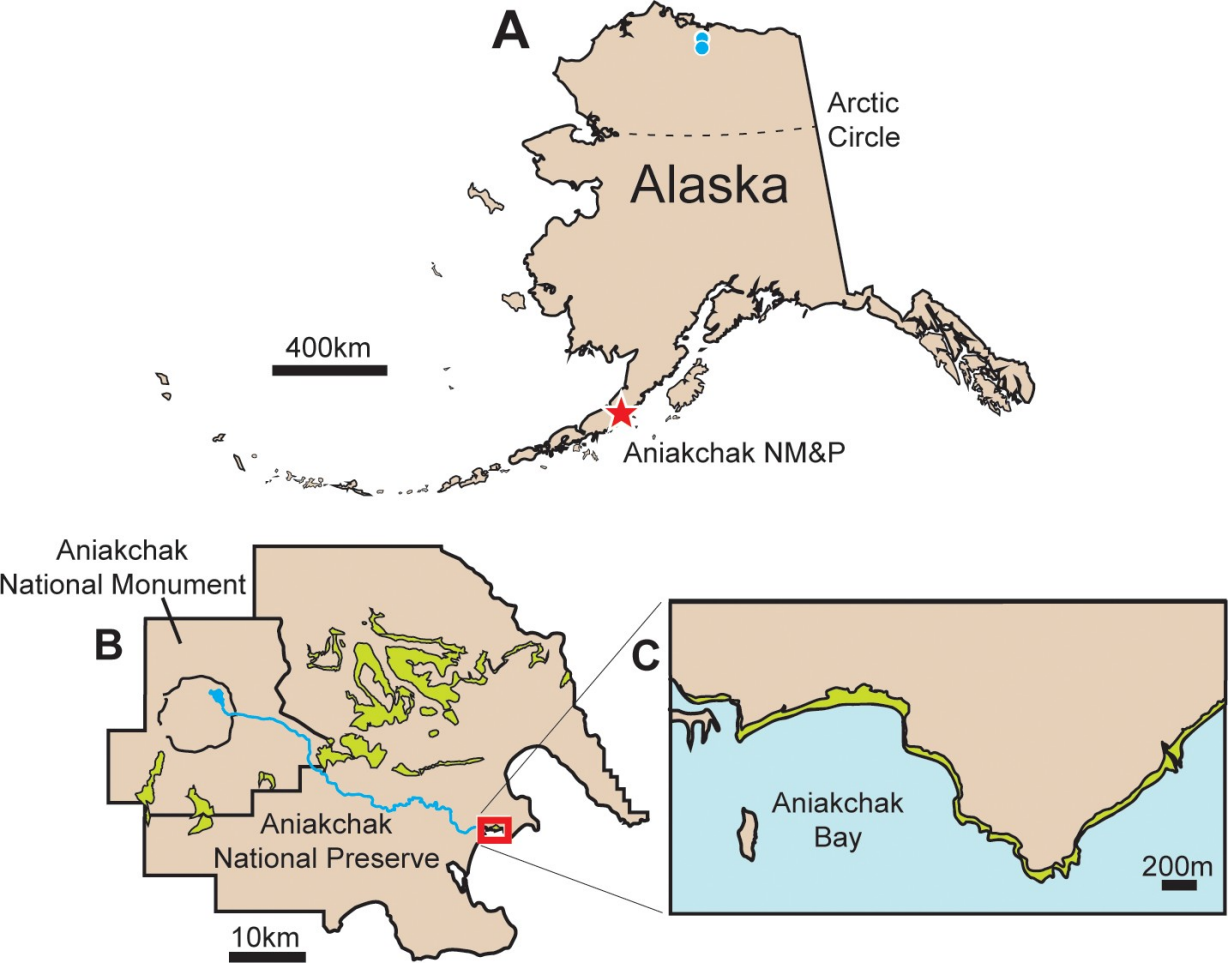
Composite figure showing location of Aniakchak National Monument. **A**, Alaska. Red star is location of Aniakchak National Park and Preserve. Blue circles show location of dinosaur bonebeds on North Slope. **B**, Drawing of Aniakchack National Park and Preserve. The outcrop pattern for the Chignik Formation is shown in light green. Red rectangle outlines this study area. **C**, Close-up diagram of study area showing Chignik Formation exposures in light green, restricted to shoreline.

Aniakchak National Monument and Preserve (ANIA), located approximately 670 kilometers southwest of the city of Anchorage, Alaska, USA, comprises approximately 240,000 hectares and is one of the least-visited parks within the United States National Park Service (Fig 1). The park was largely established in 1978 because of the 10 kilometer-wide Aniakchak Caldera, a circular geomorphic feature that is the result of the collapse of a magma chamber during the Holocene [21, 22]. The walls of the caldera range in height from a few hundred meters to over a thousand meters. The most recent eruption from within the caldera occurred in 1931 [23, 24]. In addition to this prominent volcanic feature, sedimentary rocks ranging in age from the Upper Jurassic (Naknek Formation) to Eocene (Tolstoi Formation) are also preserved in the park [25, 26]. Of these units, the Upper Cretaceous Chignik Formation (Fig 1B) has a previously poorly documented dinosaur ichnofauna [1, 27, 28].

These rocks are part of the Peninsular Terrane, the structural unit that encompasses much of southwestern Alaska. Paleomagnetic reconstructions based on the Upper Cretaceous and Lower Tertiary volcanic rocks of this terrane suggest that the sediments of the Chignik Formation were deposited at nearly their current latitude of approximately 57 degrees north [29]. In contrast, coeval sedimentary rocks of the Peninsular Terrane elsewhere in southern Alaska provide more ambiguous paleomagnetic results, suggesting depositional origin as far as 3000 km south of the present position of the rock sequence [29]. The Chignik Formation was named by Atwood [30] for rocks exposed in the vicinity of Chignik Lagoon, approximately 75 kilometers southwest of Aniakchak Bay where the current study took place. The rock unit was recognized for its coal resources as early as 1885 [31]. The formation has a maximum stratigraphic thickness of approximately 600 m in the type area of Chignik Bay [32]. The thickness varies outside the type area, thinning rapidly to the northeast and southwest [32]. The Chignik Formation is a cyclic sequence of rocks representing predominately shallow marine to nearshore marine environments in the lower part and predominately continental environments in the upper part of the section [1, 33–35].

Based on the presence of the marine bivalves, *Inoceramus balticus* var. *kunimienis* and *I*. *schmidti*, and the ammonite *Canadoceras newberryanum*, the age of the Chignik Formation was interpreted to be late Campanian to early Maastrichtian [32]. Similarly, megafloral remains from this rock unit in Aniakchak Bay include the taxon *Trapa*?, an aquatic fern with unclear taxonomic affinity [2]. This plant is reported from the section of the Prince Creek Formation that corresponds to the Campanian-Maastrichtian boundary [36, 37]. Thus its presence in the Chignik Formation in Aniakchak Bay suggests corroborative biostratigraphic significance. These data suggest then, that the Chignik Formation exposed in Aniakchak Bay is approximately correlative with dinosaur-bearing sections in the Prince Creek Formation along the Colville River of northern Alaska, and the lower Cantwell Formation of Denali National Park, in the central Alaska Range [2].

**Fig 2:**
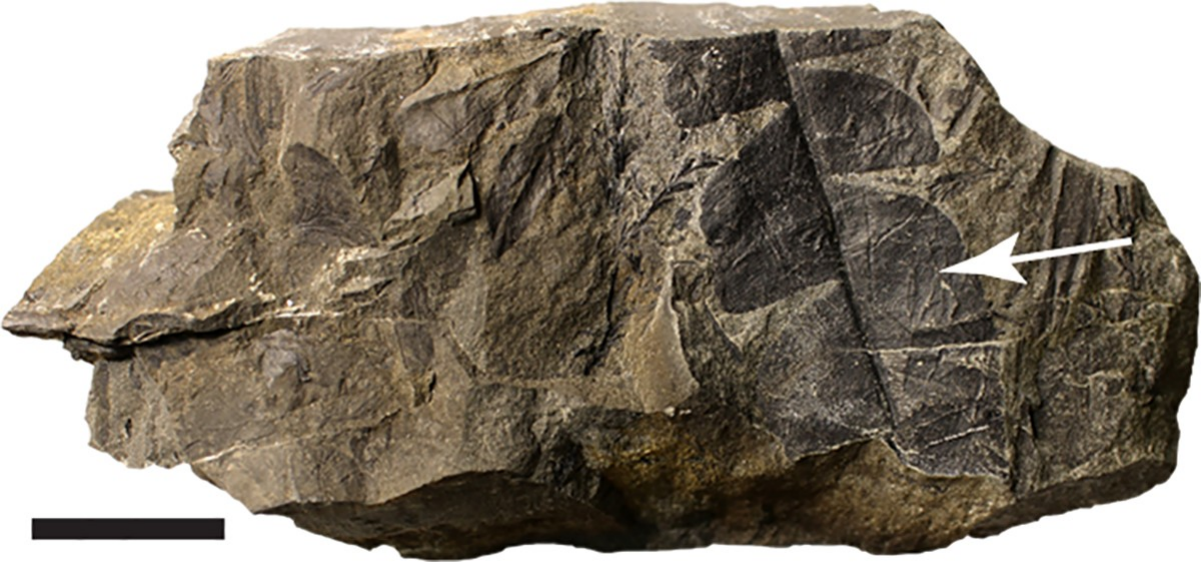
Partial *Nilssonia serotine* leaf. Leaf indicated by white arrow.

The Chignik Formation contains a rich fossil flora and the definitive work remains that by Hollick [38]. During the course of our field work, we found a leaf attributable to the cycadophyte *Nilssonia serotina* Heer (Fig 2). Modern cycads are found in subtropical to tropical environments. The presence of this fossil plant in these rocks is evidence of a much warmer environment in the Aniakchak region during the Cretaceous than is experienced there now.

**Fig 3:**
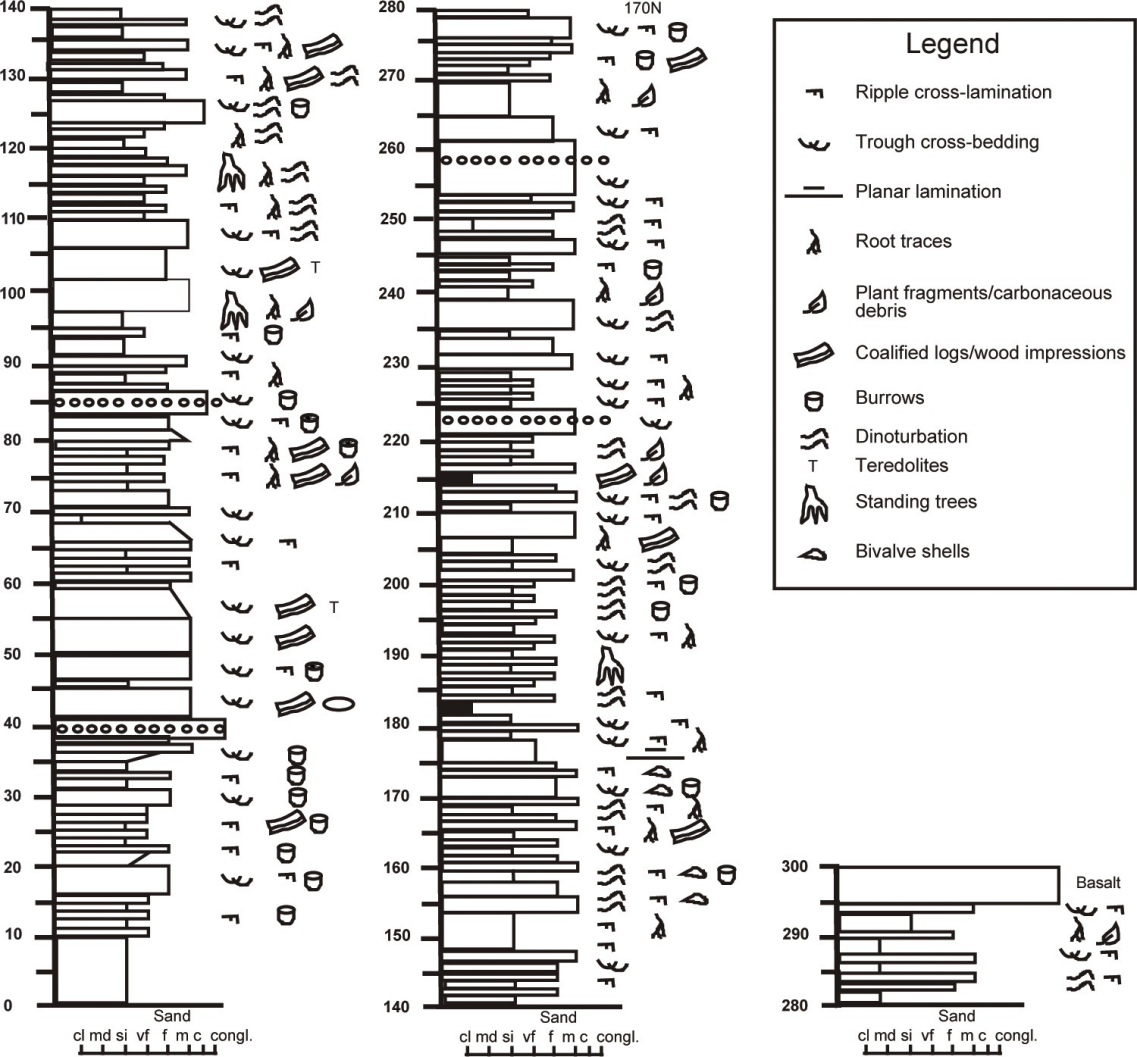
Stratigraphic section of Chignik Formation in Aniakchak Bay, Alaska, USA.

Fiorillo and Parrish [1] originally measured a 280 m section of the Chignik Formation in the study area in Aniakchak Bay. We expanded on this work by extending the section to encompass slightly more of the marine part of the section, as well as adding additional details of the sedimentology. A 300 m section was measured in detail comprising marine, coastal/tidal, and continental depositional environments (Fig 3). The basal marine part of the section (0–38 m) consists of offshore shales and siltstones, with lesser thin sandstone interbeds. These fine-grained offshore deposits coarsen upward into bioturbated, cross-bedded or horizontal laminated sandstones interpreted as regressive shoreface sandstones.

Coastal deposits primarily include tidal flat deposits [39]. Supratidal marsh environments are characterized by interbedded sandstone and mudstone, abundant root traces, and coals. Intertidal deposits consist of cross-bedded and ripple cross-laminated sandstones with siltstone interbeds. Mud drapes are common on ripple cross-laminations. Bioturbation is common [40] and root traces may be present. Asymmetrical and symmetrical ripples are present in places, and some of the ripples have flat-topped crests. Lower sand flat deposits are dominated by flaser bedding, while wavy and lenticular bedding is most common in mixed flat environments. High mudflat deposits are dominated by mudstones with some sand stringers. Tidal flat environments of the Chignik Formation show both fining and coarsening upward successions. Layers of broken bivalve shells in various orientations are noted between 155–175 m, as well as some low-angle and planar laminated sandstones which are interpreted to represent wave action along the coast. The bivalve shells were likely thrown on the tidal flats during storms where they subsequently became preserved as shell layers [41]. Tidal channels are characterized by cross-bedded and ripple cross-laminated medium to fine-grained sandstones that fine upward to very fine-sandstone, siltstone, and shale.

Continental deposits are represented by large meandering channel deposits, including well-developed, fining upward successions of cross-bedded and ripple cross-laminated sandstones and overbank deposits, including crevasse splays, crevasse channels and floodplain mudstones and coals. Root traces are pervasive throughout floodplain mudstones and crevasse splays. Organic matter is common, and plant detritus and coalified wood may also be present. Standing trees are observed rooted in the upper parts of organic-rich shales or coals in several places, which are truncated by overlying point bar sands.

Taken as a whole, the measured section along the coast in Aniakchak Bay reflects an up-section shift from shallow marine, to coastal/tidal flat, to fluvial depositional environments. The large fluvial channel that immediately overlies marine deposits contains abundant evidence of brackish water conditions including *Teredolites*-bored coalified wood, jarosite, and abundant vertical and subvertical burrows. This is followed by a thick tidal flat succession which is, in turn overlain, by nonmarine fluvial channel and overbank deposits. Tidal flats and terrestrial overbank deposits show abundant dinoturbation. The entire succession is interpreted as a transgressive-regressive sequence, consistent with a tide-dominated estuary fill [42]. As such, the modern Aniakchak River entering Aniakchak Bay provides a superb modern analog for the Cretaceous sedimentary rocks preserved here. In summary, the rocks of the Chignik Formation are marine in the lowermost part of the section and becomes more terrestrial in nature moving up section. The section represents deposition in shallow marine to distal coastal plain settings.

### Tracks and track makers

Dozens of dinosaur tracks have now been discovered from the coastal exposure of the Cretaceous Chignik Formation in ANIA (Fig 1C). Tracks were found in cross-section within the face of cliffs and in planar view either on in situ bedding planes or on isolated eroded blocks that had fallen from the cliff face. Tracks were photographed, measured, coordinates recorded, and molds were made of select, representative tracks. The molds and casts made from them are housed at the Perot Museum of Nature and Science (DMNH) in Dallas, Texas, and these serve as physical voucher specimens. Specimen numbers are provided in this text for casts of tracks. In cases in which only photogrammetry was used to record and reconstruct tracks, field numbers are provided in the text. In addition, the photographic models of footprints 16FP-04 (large hadrosaur), 16FP-10 (ankylosaur), 16FP-15 (small hadrosaur), and 17FP-03 (large theropod) were constructed with Agisoft Metashape professional software (ver.1.5.0), using 65, 47, 46, and 11 images, respectively. The photographs for 3D models were taken with a Nikon D3100 camera (resolution 4608 x 3072). Contour maps of these images were made using 3D landscaping software Kashmir 3D (ver. 8.9.8 Beta). The contour intervals of 16FP-04 (large hadrosaur) and 16FP-10 (ankylosaur) are 2.0 mm, and those of 16FP-15 (small hadrosaur) and 17FP-03 (large theropod) are 1.0 mm.

### Hadrosaurid tracks

**Fig 4:**
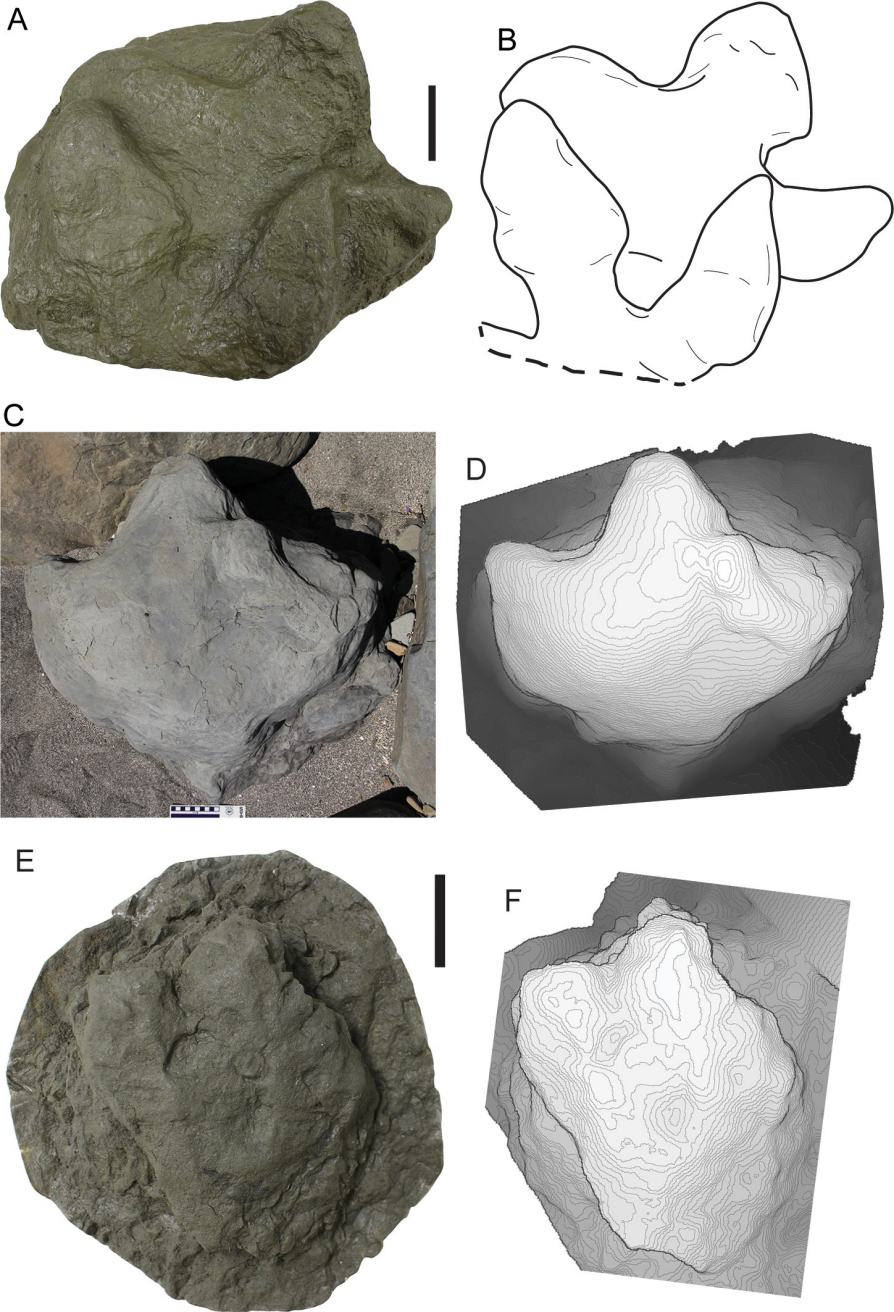
Representative hadrosaur tracks. **A**, cast of DMNH 2016-05-03, a pair of large, overlapping hadrosaur tracks; **B**, outline drawing of tracks in A; **C**, photograph of 16FP-04, large hadrosaur track; **D**, photogrammatic contour map of 16FP-04; **E**, cast of DMNH 2016-05-05, a small hadrosaur track; **F**, photogrammatic contour map of DMNH 2016-05-05 generated from photos taken in the field. Scale bars in A through C equal 10cm. Scale bar in E and F equals 5cm.

The most abundant tracks found in this study can be attributed to hadrosaurid dinosaurs (Fig 4). The only set of manus-pes impressions found are the set originally described by Fiorillo and Parrish [1], and all other hadrosaur tracks are pes impressions. The pes tracks are tridactyl, wider than long, with digits that are wide, short, and terminate bluntly. The pes tracks also have wide, bi-lobed, posterior margins. The tracks are preserved in concave epirelief, and convex epirelief.

**Fig 5:**
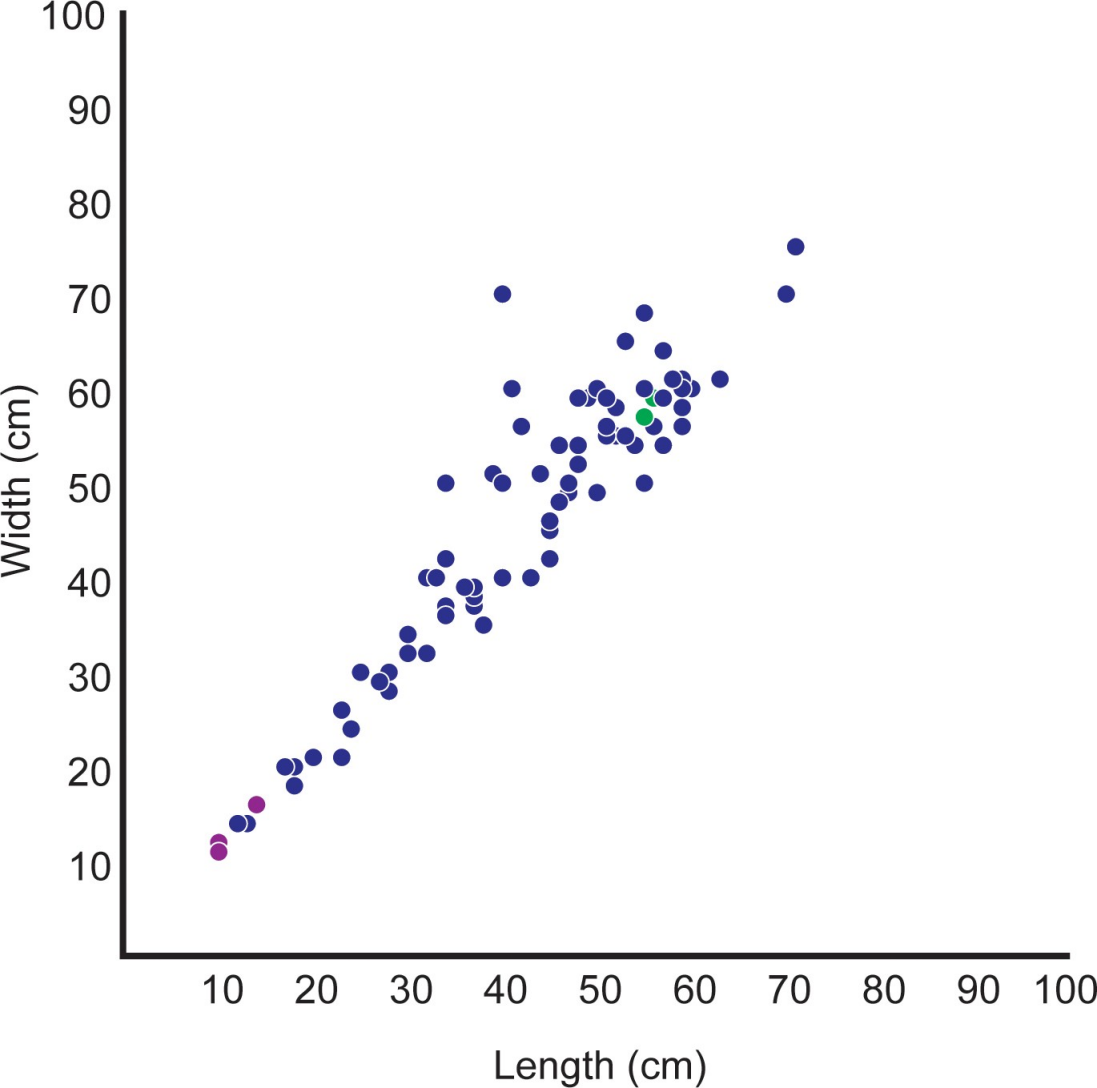
Graph of length-width distribution of all hadrosaur tracks found in ANIA to date. Green plot-points represent the original tracks found in 2001. Blue plot-points represent tracks found in 2016–2018 field seasons.

The morphological features of these tridactyl tracks allow attribution to hadrosaurs, and specifically the ichnogenus *Hadrosauropodus* isp. [43–45]. This ichnogenus was also recorded in the correlative lower Cantwell Formation of Denali National Park in the central Alaska Range [2, 27]. Further, the divarication angles between digits II and IV of these tracks range from 62° to 101°, angles that fall within the range of previously published divarication angles for tracks attributed to hadrosaurs [43, 46]. The tracks range in size to encompass tracks made by juveniles, subadults, and adults (Fig 5). The range in track size covers all the four stages of hadrosaur track sizes observed and described from a large hadrosaur tracksite in Denali National Park [7].

### Ankylosaur tracks

**Fig 6:**
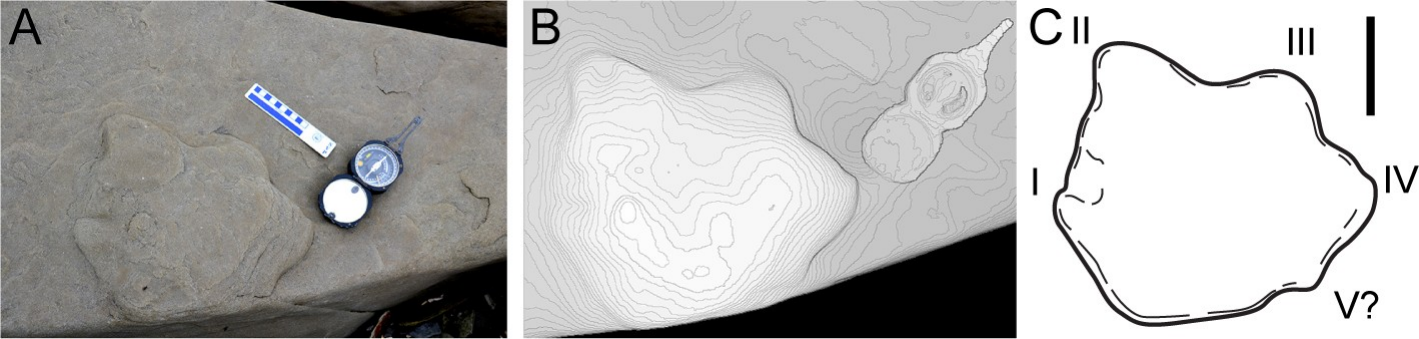
DMNH 2016-05-07, attributed to the ankylosaur ichnotaxon *Tetrapodosaurus*. **A**, photograph of specimen in field; **B**, photogrammatic, 3 dimensional contour map of specimen; **C**, outline drawing of track. Roman numerals in C indicate tentative digit identification. Scale bars equal 10cm.

Two isolated tracks, each from fallen blocks, were discovered that we attribute to thyreophorans and most likely ankylosaurs. These tracks were preserved in concave epirelief (Fig 6). Both tracks are wider than long; one track has a length of 14.5 cm and a width of 19.5 cm, while the other track has a length of 29.5 cm and a width of 35 cm. Each track preserves five digits, indicating these to be manus tracks rather than four-digit pes tracks. Digits I and V are significantly reduced in size compared to digits II-IV.

The morphology of ceratopsian and thyreophoran feet is very similar. In their overview of the global distribution of purported ankylosaur tracks, McCrea et al. [47] provided some perspectives on the skeletal and footprint characters distinguishing ankylosaur tracks from ceratopsian tracks. McCrea et al. [47] pointed out a significant difference in that ankylosaurs had proportionately longer toes when compared to metatarsals, while in ceratopsians the relationship is reversed, with metatarsals longer than the toes. They inferred from this that ankylosaur tracks have well developed toe impressions when compared to ceratopsian tracks [47]. McCrea et al. [47] also submitted that often digit I is the most prominently expressed digit in manus tracks for the ankylosaur ichnogenus *Tetrapodosaurus*. However, while that pattern may be common, in the original description of this ichnogenus by Sternberg [48], digit I was not prominently expressed in any of the figured tracks, so that character is not necessarily defining. Regardless, in ankylosaur manus prints the distribution of digit impressions is arcuate, resulting in a star-shaped track [47–50]. In light of these previous works, the two tracks from ANIA described here are attributed to the ichnogenus *Tetrapodosaurus*.

### Non-avian theropod track

**Fig 7:**
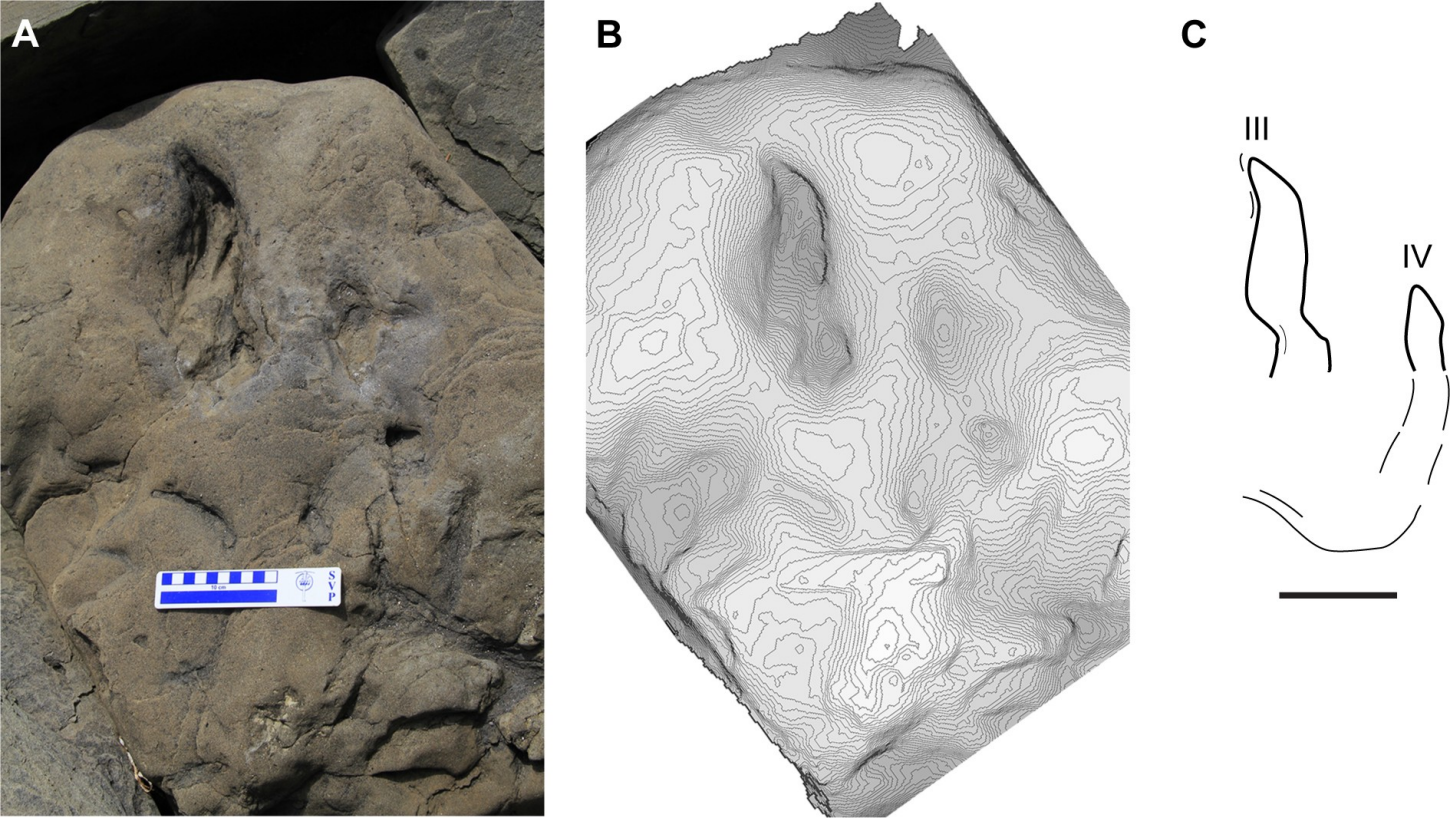
Large tridactyl track (Field #17AF7-14-1), attributed to the theropod ichnogenus *Grallator*. **A**, photograph of specimen in the field; **B**, photogrammatic 3 dimensional contour map of the specimen in A; **C**, outline drawing of 17AF7-14-1. Roman numerals in C indicate tentative pedal digit identification. Scale bars equal 10cm.

A single large tridactyl track was found (Fig 7). The track is preserved in concave epirelief. The digits are long and thin, and they taper to a point. In addition to the sharply terminated distal end, digit III also has a slight sinusoidal curve. The lengths of digits II, III, and IV are respectively, 22, 31, and 23 cm respectively. The morphology of this track allows attribution as a medium to large non-avialan theropod. Given the track length, using an equation of 4X the track length as an estimate of hip height, and 3.75X the hip height as an estimate of body length [10, 51, 52], this track was made by a non-avialan theropod approximately 4.5–5 meters long.

There are a great number of tridactyl non-avian theropod ichnotaxa, and rather than address all of the nuances distinguishing these taxa, here we provide a subjective comparison of this track to two of the more commonly discussed ichnotaxa, *Grallator* and *Eubrontes*. A number of authors have attempted to quantify the differences between the two ichnogenera *Grallator* and *Eubrontes* [53–55]. While techniques for comparisons have varied, the most significant aspect distinguishing these two ichnogenera is the relative length of digit III in relation to digits II and IV, and the track length as a whole. Smith and Farlow [55] relied more on simple ratios (Table 1) between digits III and II, and digits III and IV. Comparison of the digit III/II and III/IV length ratios for the Aniakchak track described here with those provided by Smith and Farlow [55] shows the Alaska track to have a greater affinity with *Grallator* than with *Eubrontes*. The overall morphology of the Aniakchak track fits comfortably, then, within the attributes of *Grallator* isp.

**Table 1:** Average ratios of digit lengths for *Grallator* and *Eubrontes* based on original sample data (17 and 16 tracks, respectively) from Smith and Farlow [54], as well as the ratios for the Aniakchak track (ANIA) described here. Note that the ratios indicate that digit III is proportionately longer in *Grallator* than in *Eubrontes*. Based on these ratios, the Aniakchak track is assigned to the ichnogenus *Grallator*.

Taxon	III:II	III:IV
*Grallator*	1.44	1.22
*Eubrontes*	1.11	0.93
*Grallator* (ANIA)	1.41	1.35

### Large avian theropod tracks

**Fig 8:**
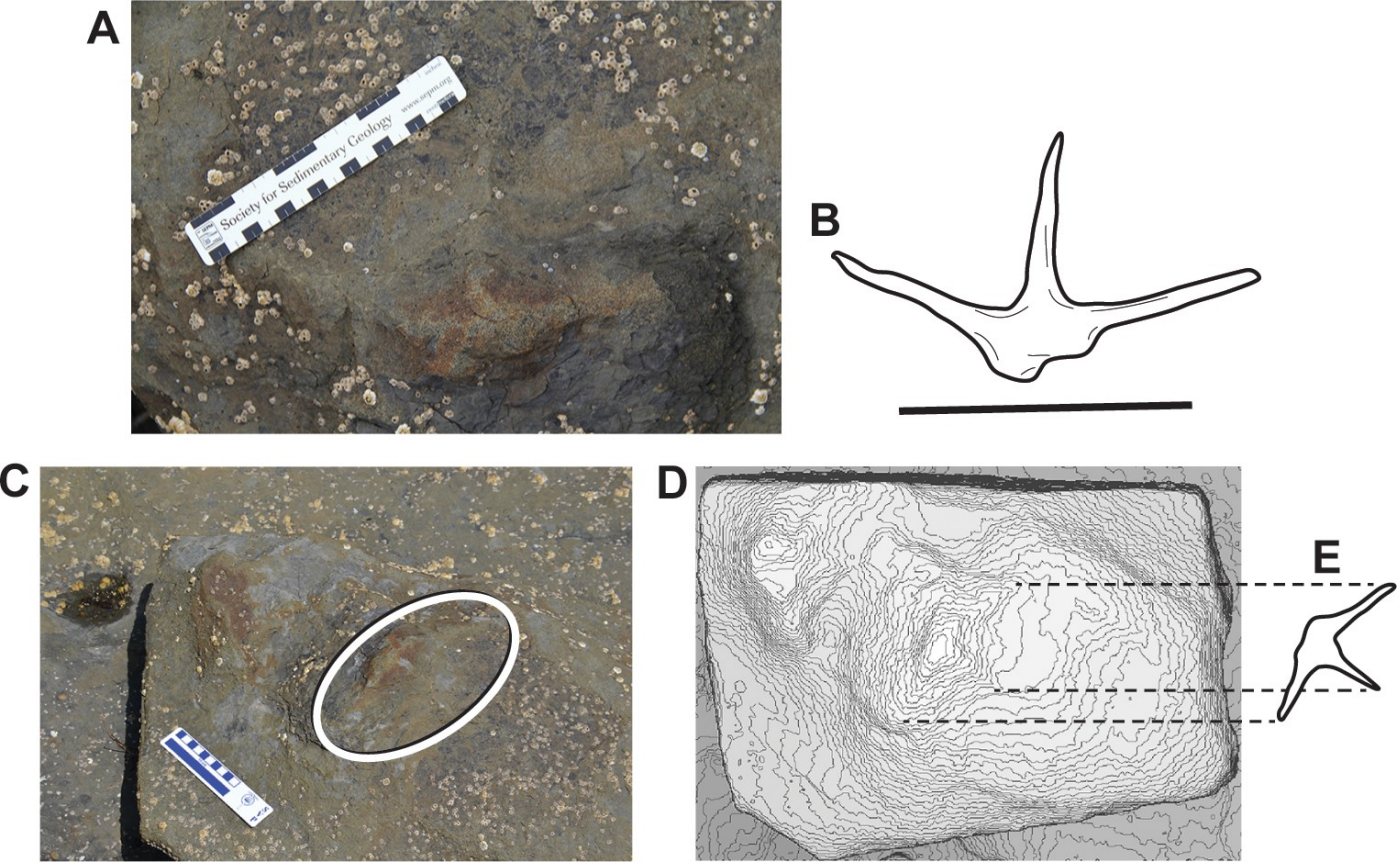
Field #17AF7-15-2, large avian theropod track, attributed to the ichnotaxon *Magnoavipes*. **A**, photograph of specimen in the field, in planar view, anterior to top of image; **B**, outline drawing of track in A; **C**, photograph of same track in field, taken from lower, oblique angle. Ellipse encompasses the track; **D**, photogrammatic 3 dimensional contour map of the track, in approximately the same view as in C; **E**, outline drawing of track in D, with dashed lines indexing to tips of digits in D. Scale bars equal 10cm.

A paired set of bird-like tridactyl tracks with long, slender toes are attributed to a large, avian theropod (Fig 8). Both tracks are approximately 30% wider than they are long, with length measurements of 13.5 cm, and slightly different width measurements of 18.5 cm and 19.0 cm wide. The tracks have divarication angles between digits II-IV of 150° and 146° respectively. No hallux (digit I) impressions are preserved. The wide divarication angles, and the slender proportions of the digits clearly indicate an avian trackmaker.

Lee [56] recognized large, aviform, tridactyl tracks with very slender pedal digits and sharp terminations in the Woodbine Formation (middle Cenomanian) of Texas. These tracks ranged from 19 to 21 cm in length, were wider than long, and had divarication angles from 109° to 118°. Given the overall size of these Texas tracks, Lee [56] erected the ichnogenus *Magnoavipes*. While the tracks from ANIA are somewhat smaller than those described by Lee [56], other attributes of the Alaskan tracks are similar, such as: slender digits, tracks wider than long, digits that terminate sharply, and a wide divarication angle for digits II-IV. Together, these features allow attribution of these tracks from ANIA to the ichnogenus *Magnoavipes* isp. Further, these ANIA *Magnoavipes* tracks are very similar to the tracks of modern cranes such as Sandhill Cranes (*Antigone canadensis*; e.g., [57]) and Common Cranes (*Grus grus*; e.g., [58]), a point not lost on Lee [56] who suggested that *Magnoavipes* tracks were made by a Cretaceous bird with crane-like proportions. These two extant cranes have divarication angles for digits II-IV that range from 120° to 122°, and 120° to 123° respectively. The divarication angles of the Aniakchak tracks, while greater than those reported from *Magnoavipes lowei* (109°-118°) and *Magnoavipes denaliensis* (110°) [5, 56], and much greater than the somewhat dissimilar *Magnoavipes caneeri* (85°) are still within the upper range of such angles found in modern birds. Our comparisons with Lee’s original description [56], the definitions put forth by De Valais and Melchor [59] for avian tracks in general, and with the published work on modern birds allows us to confidently attribute these fossil footprints to an avian theropod (bird). While there is some question regarding the affinities of the trackmaker of *Magnoavipes* (see Supplemental files and figures), it remains that these ANIA tracks best fit within this ichnogenus.

### Small avian theropod tracks

**Fig 9:**
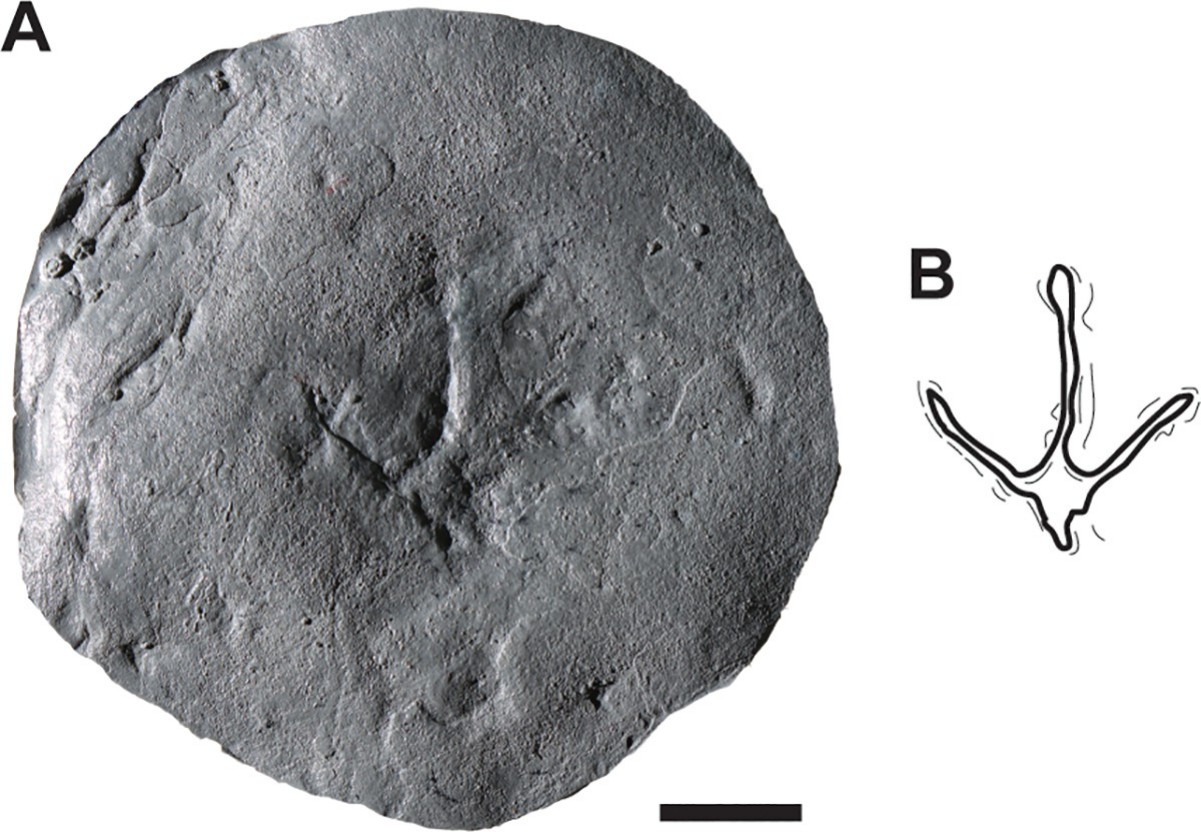
DMNH 2018-12-01, small avian track attributed to the ichnogenus *Aquatilavipes*. A, photo of cast of track. B, outline drawing of track. Scale bar equal 2cm.

Currently, only a single track can be attributed to a small, avian theropod. This impression has three slender toe impressions (Fig 9). No hallux (digit I) impression is preserved. The track is approximately 4.5 cm long and 4.5 cm wide. Digit III is the longest and digit IV is approximately the same length as digit II. The divarication angle between digits II and IV is 85°. The morphology of this fossil track compares well with the *Aquatilavipes swiboldae* [60]. Though reported in Asia [61–63], this ichnotaxon is found largely in Cretaceous units throughout western North America [5, 60, 64, 65].

## Discussion

**Fig 10:**
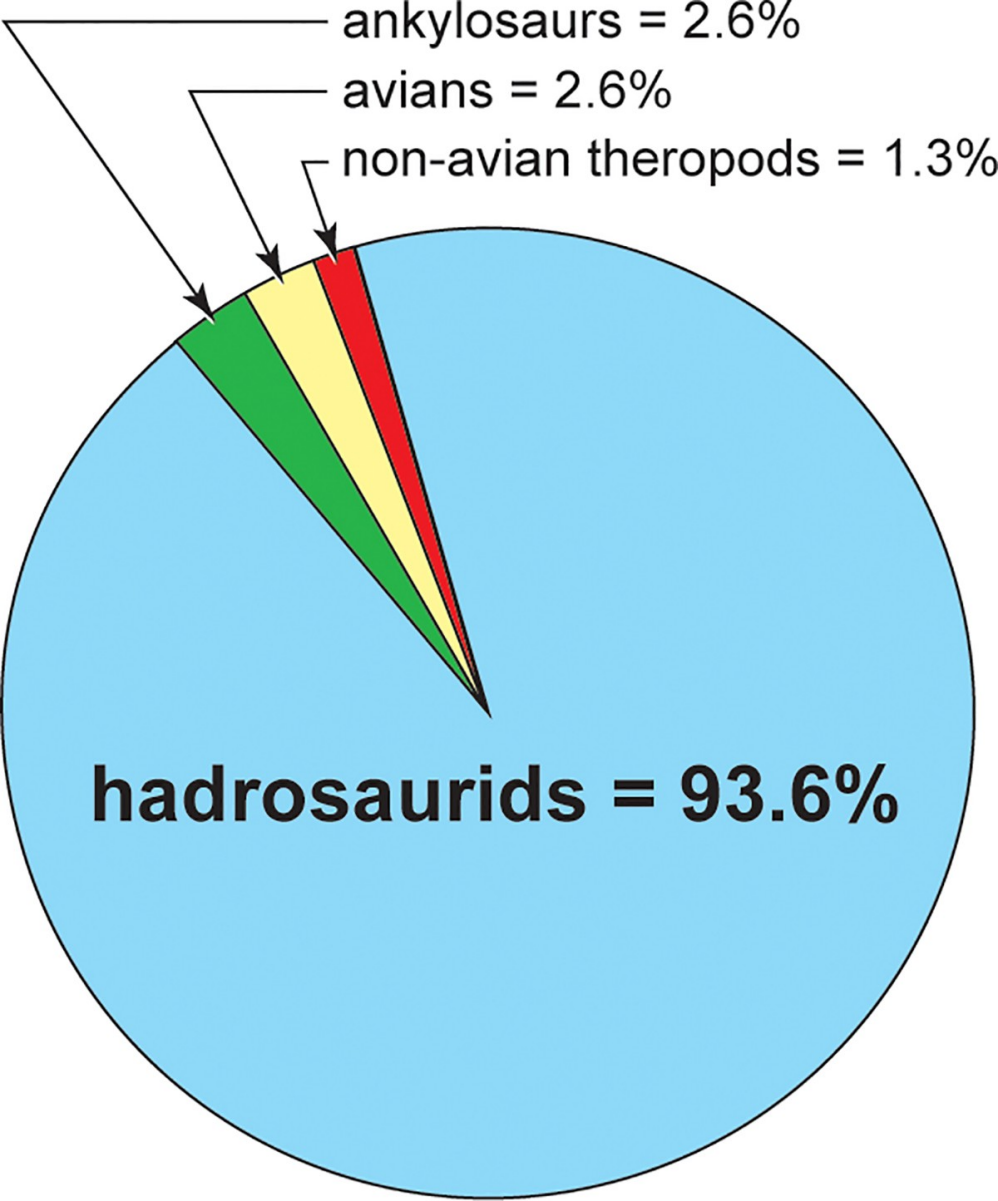
Dinosaur track diversity in ANIA. Pie chart showing the relative abundances of the dinosaurian ichnotaxa found in the Chignik Formation. Light blue = hadrosaurids. Green = ankylosaurs. Yellow = avians. Red = non-avian theropods.

The paleontological survey work in Aniakchak National Monument from 2001–2002, and 2016–2018 has now revealed 74 separate dinosaurian track sites, dramatically increasing the dinosaur track record from the Alaska Peninsula. While the tracks record small and large avialan theropods, large non-avialan theropods, and ankylosaurs, 93% of all tracks found in these coastal Chignik Formation exposures thus far can be attributed to hadrosaurian dinosaurs (Fig 10). Some of these clades bear further discussion.

Several workers have [7, 66, 67], used the size distribution of hadrosaur tracks found in Cretaceous rocks of the central Alaska Range, South Korea, and Gobi Desert, respectively, to speculate about the population structure in these dinosaurs, taxa that have been generally accepted as gregarious. Their studies suggested that hadrosaurs went through a period of rapid growth during adolescence. Further, because their data were similar to the data in the Alaska sample, Nakajima et al. [67], suggested that population structure within herds of hadrosaurs were similar independent of latitude or continent. The size distribution of hadrosaur tracks from ANIA (Fig 5) supports the hypothesis that population structure of hadrosaurs across latitudes was similar.

The discovery of dinosaurs in the Arctic initially puzzled researchers and one of the early ideas to explain these discoveries in such extremely seasonal latitudes was that these Arctic dinosaurs must have undergone large-scale migrations to cope with the extreme nature of the ancient high-latitude environment [2]. While it is no longer thought that hadrosaurs survived the winter using seasonal migrations similar to caribou [7, 68, 69], evidence does suggest that dinosaurs migrated between what is now modern Asia and North America through Alaska during the Cretaceous [6, 11, 70–73].

As explained earlier, the Chignik Formation records a cyclic succession of sedimentary rocks representing shallow marine environments in the lower part and predominantly non-marine environments in the upper part. Further, the non-marine environments primarily record deposition on an ancient alluvial-deltaic coastal plain that was dominated by sinuous meandering fluvial channels, with abundant crevasse splays, small lakes and ponds, and a few thin peat swamps. There is also evidence of tidal influence on some of the distal deposits, including tidal flats as well as marginal marine beach and estuarine deposits.

The well-known dinosaur remains in the Prince Creek Formation, approximately 1,500 kilometers farther north in Alaska, overlap in age with the dinosaur discoveries in the Chignik Formation [1, 2, 17]. A recent study [20] integrated detailed depositional processes with the vertebrate body fossil record within the Prince Creek Formation paleoecosystem of the North Slope of Alaska. That study showed a pattern of dominance of hadrosaur remains, specifically bones and teeth of *Edmontosaurus*, in the more distal basin represented by lower delta plain facies. In contrast, ceratopsian (*Pachyrhinosaurus*) remains were more prevalent in the more proximal part of the basin, represented by better drained coastal plain facies. The results of this interdisciplinary study in the Chignik Formation shows a similar pattern based on dinosaur track distribution and frequency. It suggests the same habitat preference model for Cretaceous high-latitude hadrosaurs of ANIA as proposed for the hadrosaurs of the Prince Creek Formation. That is, hadrosaurs preferred areas that included lowland deltas and other tidally influenced habitats.

This understanding of hadrosaurs’ habitat preference allows questions on how specific habitats might change through time and space. Continued fine-tuning of our understanding of the details of these habitat preferences will not only illuminate the potential causal mechanisms for biogeographic patterns across Cretaceous Beringia, but also tell us something about large-scale ecosystem processes through deep geologic time. In other words, understanding how specific habitats change through time will inform the ‘how, when, and why’ dinosaurs migrated across the Beringian land bridge.

**Fig 11:**
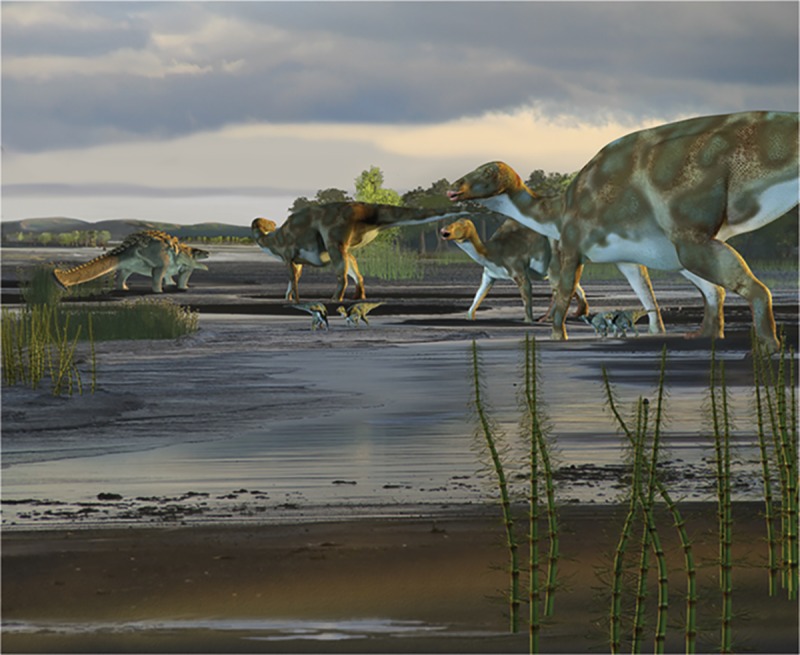
Artistic rendering of ANIA in Late Cretaceous. Based on sedimentological and ichnological findings of this study. Reprinted from [28] under a CC BY license, with permission from [Karen Carr], original copyright [2018].

We cannot determine with certainty to which ankylosaurian clade the makers of the ankylosaur tracks described here belonged. However, Butler and Barrett [74] used a global database of Cretaceous dinosaurian herbivores to study possible associations between broad sedimentary settings and dinosaur taxa. They showed that nodosaurids preferred coastal settings while ankylosaurids preferred more upland environments. The sedimentology of the rocks in this study area clearly shows these sediments were deposited as part of a coastal paleoenvironment (Fig 11). If the determinations of Butler and Barrett [74] are accurate, it is reasonable to suspect that these ANIA ankylosaurian tracks were more likely made by nodosaurids than more proximal upland-preferring ankylosaurids.

The presence of fossil bird tracks in the Cretaceous rocks of Alaska as key components of the vertebrate biodiversity of the ancient Arctic was well established through the work on the correlative lower Cantwell Formation of Denali National Park [5]. That work provided evidence of at least seven types of fossil birds in the Cantwell Formation, as shown from fossil tracks. Among those ichnogenera is *Aquatilavipes* and *Magnoavipes*, thus the ANIA footprints in this report extends the known geographic ranges of these ichnogenera in the ancient Arctic.

## Conclusions

While there are now numerous records of dinosaurs from Cretaceous rocks around the state of Alaska, very few fossil records of terrestrial vertebrates are known from the Mesozoic rocks of the southwestern part of the state. This study documents the extensive occurrences and diversity of the over 75 new dinosaur tracksites, from exposures of the Cretaceous Chignik Formation in Aniakchak National Monument of the Alaska Peninsula, thereby dramatically increasing the dinosaur record from this region. These tracks are in the Late Cretaceous (Maastrichtian) Chignik Formation, a cyclic sequence of rocks, approximately 500–600 m thick, representing shallow marine to nearshore marine environments in the lower part and continental alluvial coastal plain environments in the upper part of the section. This rock unit was deposited at approximately its current latitude which is almost 57° N.

Most of the combined record of tracks can be attributed to plant-eating duck-billed dinosaurs, with a track size range corresponding to full-grown adults to juveniles. Other tracks are attributed to armored dinosaurs, meat-eating dinosaurs, and two kinds of fossil birds. The larger bird tracks are attributed to *Magnoavipes*, a crane-sized bird, while the smaller bird tracks attributed to a bird about the size of a modern Willet. The track size of the predatory dinosaur suggests a body size approximately 6–7 m long, about the size of *Nanuqsaurus*, the tyrannosaurid known from bones from the North Slope.

Previous interdisciplinary sedimentologic and paleontologic work in the correlative and well-known dinosaur bonebeds of the Prince Creek Formation 1400km-1500km further north in Alaska suggested that high-latitude hadrosaurs preferred distal coastal plain or lower delta plain habitats. The current interdisciplinary paleontologic and sedimentologic project in the Chignik Formation finds that hadrosaur tracks here were also made in distal coastal and delta plain conditions. This similarity may corroborate the habitat preference model for Cretaceous high-latitude dinosaurs proposed for the data gathered from the Prince Creek Formation.

## Supporting information

S1 Fig Size of Magnoavipes tracks compared to unequivocal bird tracks. Graph showing published bird track sizes (mean print length in centimeters) of 29 large modern birds, three Cenozoic birds, and the Cretaceous ichnotaxon *Magnoavipes*. Black bars indicate modern, extant species. Gray bars indicate modern but extinct moas. Three sets of tracks from large Cenozoic birds are shown by tan bars. *Magnoavipes lowei* from the Cenomanian of Texas, and *M*. *denaliensis* from the Campanian-Maastrichtian of Alaska are depicted with light green bars.
(TIF)

S2 Fig Outline drawings of tridactyl theropod tracks. A, non-avian theropod footprint from Early Cretaceous Glen Rose Limestone, Texas. B and C moas [28, 29]. D, modern Sandhill Crane [22]. E, *Magnoavipes* [1]. Note greater similarity between A, B, and C than to D and E, and greater similarity between D and E than to flightless non-avian and avian theropods. All tracks displayed to same overall length.
(TIF)

S3 Fig Range of avian pedal digit II-IV divarication angles. Graph shows pedal digit II-IV divarication angles (in degrees) of 35 modern birds based on published images, shown with solid black bars. Divarication angles for *Magnoavipes lowei* and *M*. *denaliensis* are shown by light green bars. Length of thick bars indicates the range of measured angles for the tracks of each taxon. Lower, white-background part of graph indicates the range of divarication angles for tracks typically considered to be made by ‘non-avian theropods’. Pink background indicates part of graph with divarication angles typically accepted as being made by avian theropods (birds).
(TIF)

S4 Fig Three examples of modern bird trackways. A, tracks of a Common Grackle (ratio of FL/PL = 0.24); B, tracks of a Spotted Thick-knees (ratio of FL/PL = 0.19) Reprinted from [26] under a CC BY license, with permission from [Chris & Mathilde Stuart], original copyright [2013].; C, tracks of a Ring- necked Pheasant (ratio of FL/PL = 0.16) Reprinted from [27] under a CC BY license, with permission from [Donald McLeod], original copyright [2012]. FL = Foot Length. PL = Pace Length.
(TIF)

S1 File (DOCX)
