# Supplementary figures and images for: Dinosaur ichnology and sedimentology of the Chignik Formation (Upper Cretaceous), Aniakchak National Monument, southwestern Alaska; Further insights on habitat preferences of high-latitude hadrosaurs
Source: PLoS One. 2019 Oct 30;14(10):e0223471. doi: 10.1371/journal.pone.0223471 (PMC6821036; doi:10.1371/journal.pone.0223471)

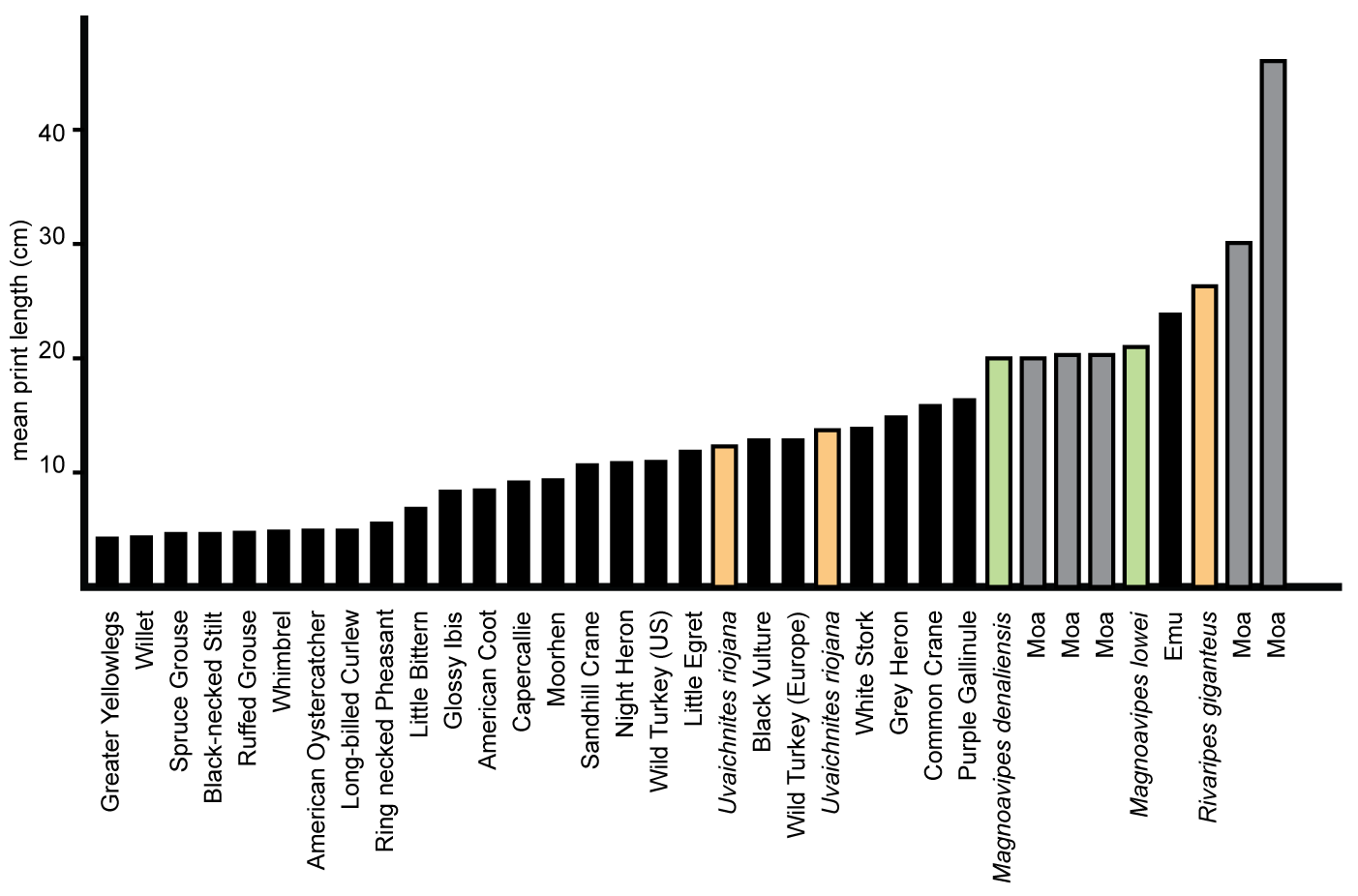

Supplement: S1 Fig — Graph showing published bird track sizes (mean print length in centimeters) of 29 large modern birds, three Cenozoic birds, and the Cretaceous ichnotaxon Magnoavipes. Black bars indicate modern, extant species. Gray bars indicate modern but extinct moas. Three sets of tracks from large Cenozoic birds are shown by tan bars. Magnoavipes lowei from the Cenomanian of Texas, and M. denaliensis from the Campanian-Maastrichtian of Alaska are depicted with light green bars. (TIF) [file pone.0223471.s001.tif]

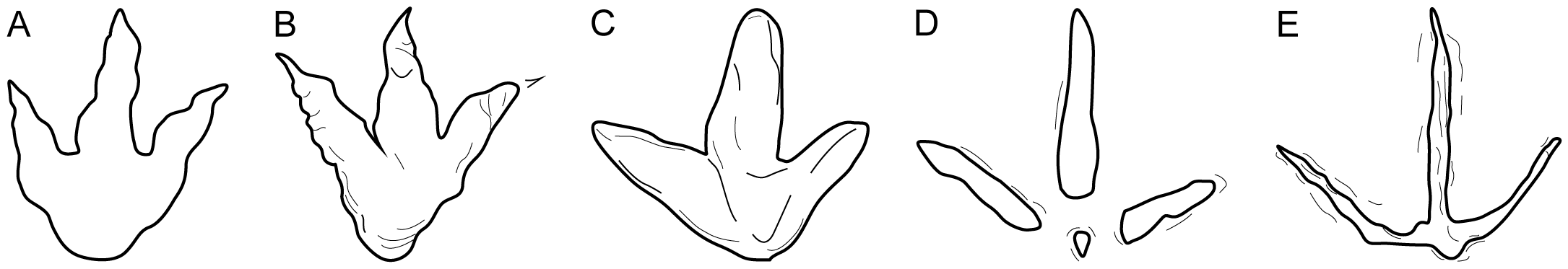

Supplement: S2 Fig — A, non-avian theropod footprint from Early Cretaceous Glen Rose Limestone, Texas. B and C moas [28, 29]. D, modern Sandhill Crane [22]. E, Magnoavipes [1]. Note greater similarity between A, B, and C than to D and E, and greater similarity between D and E than to flightless non-avian and avian theropods. All tracks displayed to same overall length. (TIF) [file pone.0223471.s002.tif]

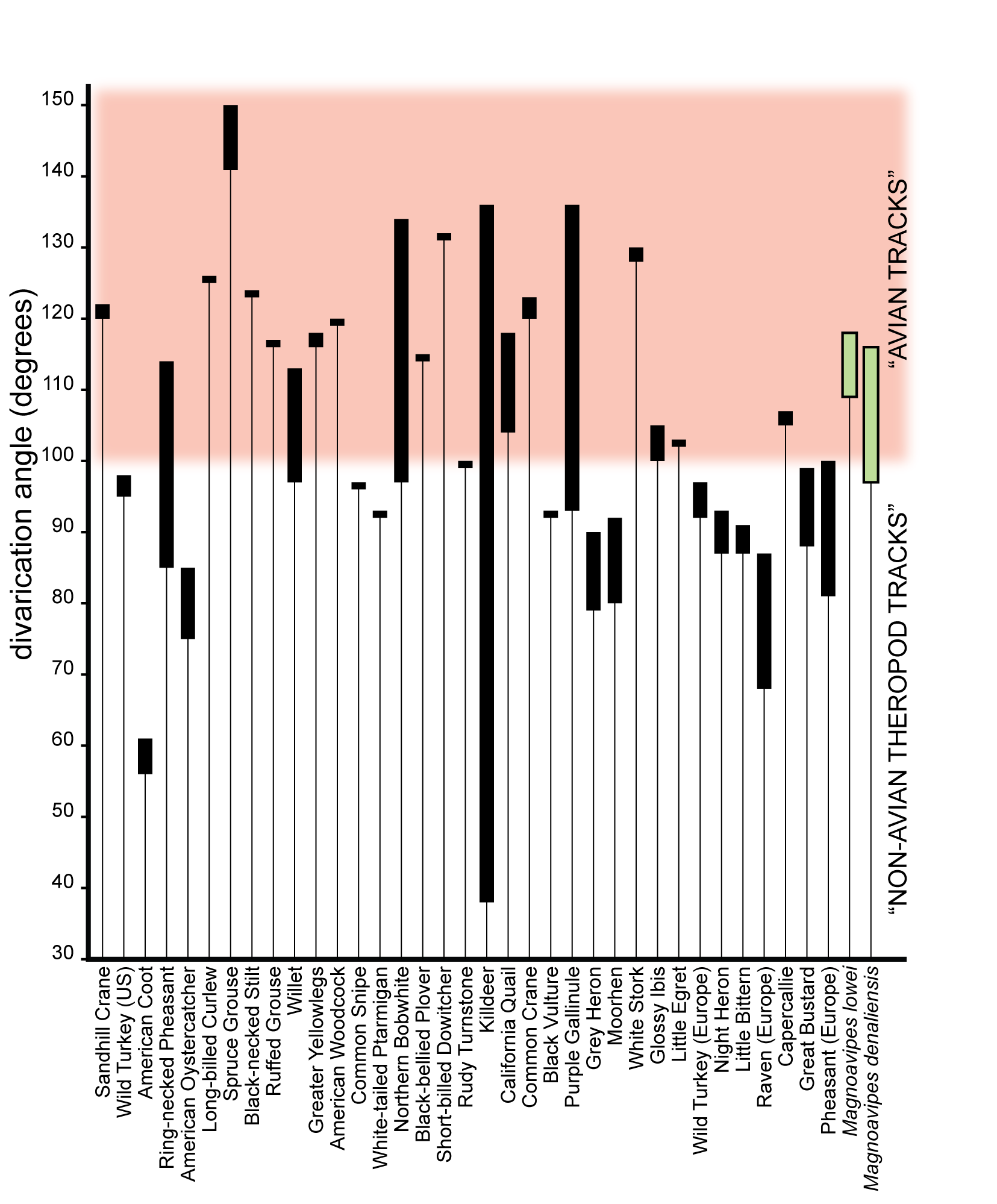

Supplement: S3 Fig — Graph shows pedal digit II-IV divarication angles (in degrees) of 35 modern birds based on published images, shown with solid black bars. Divarication angles for Magnoavipes lowei and M. denaliensis are shown by light green bars. Length of thick bars indicates the range of measured angles for the tracks of each taxon. Lower, white-background part of graph indicates the range of divarication angles for tracks typically considered to be made by ‘non-avian theropods’. Pink background indicates part of graph with divarication angles typically accepted as being made by avian theropods (birds). (TIF) [file pone.0223471.s003.tif]

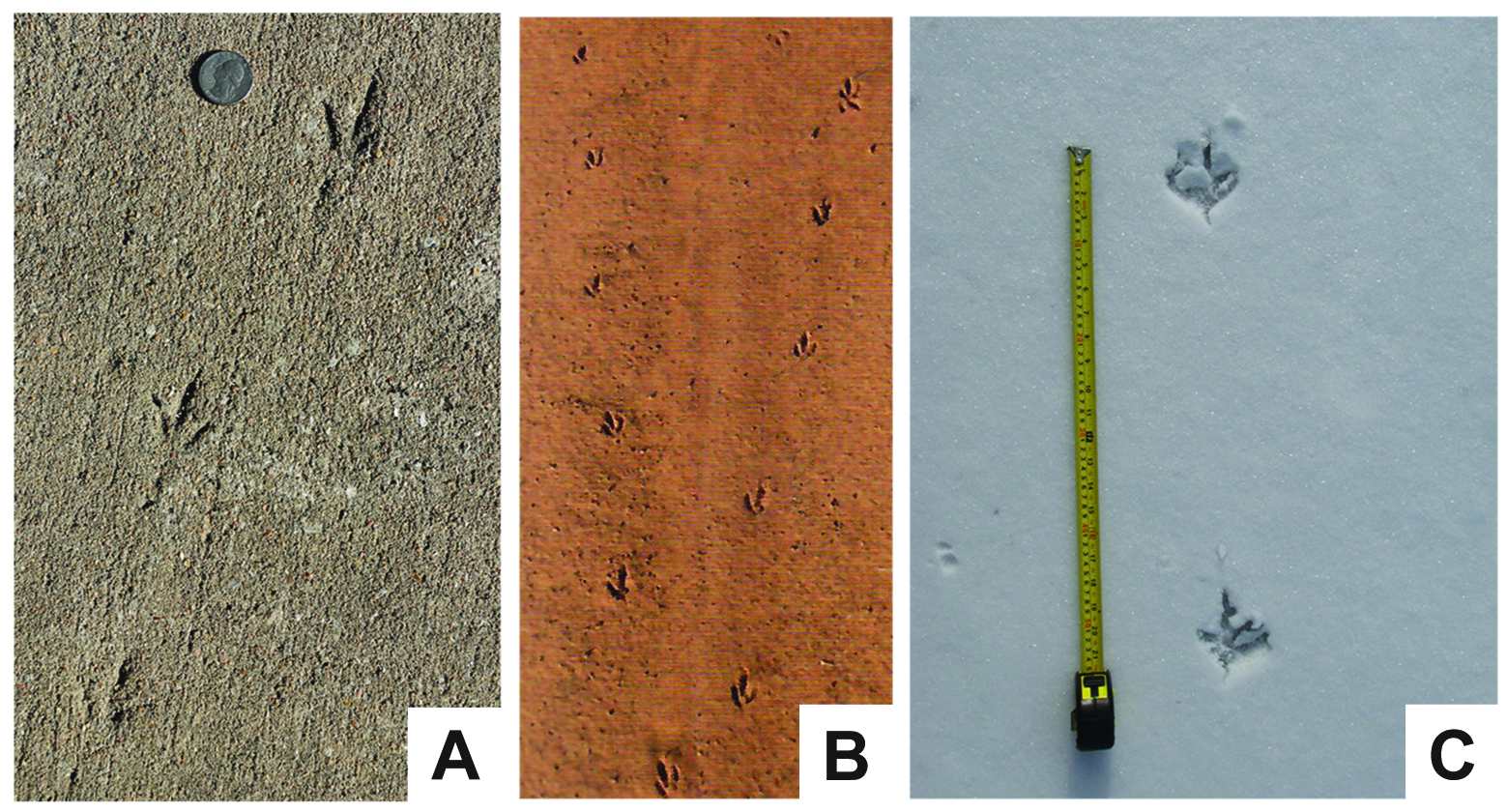

Supplement: S4 Fig — A, tracks of a Common Grackle (ratio of FL/PL = 0.24); B, tracks of a Spotted Thick-knees (ratio of FL/PL = 0.19) Reprinted from [26] under a CC BY license, with permission from [Chris & Mathilde Stuart], original copyright [2013].; C, tracks of a Ring- necked Pheasant (ratio of FL/PL = 0.16) Reprinted from [27] under a CC BY license, with permission from [Donald McLeod], original copyright [2012]. FL = Foot Length. PL = Pace Length. (TIF) [file pone.0223471.s004.tif]
