# Supplementary material for: Dinosaur ichnology and sedimentology of the Chignik Formation (Upper Cretaceous), Aniakchak National Monument, southwestern Alaska; Further insights on habitat preferences of high-latitude hadrosaurs
Source: PLoS One. 2019 Oct 30;14(10):e0223471. doi: 10.1371/journal.pone.0223471 (PMC6821036; doi:10.1371/journal.pone.0223471)
Supplement: S1 File — (DOCX) [file pone.0223471.s005.docx]

S1 Text.

**Dinosaur ichnology and sedimentology of the Chignik Formation (Upper Cretaceous), Aniakchak National Monument, southwestern Alaska; further insights on habitat preferences of high-latitude hadrosaurs**

Anthony R. Fiorillo^1*^, Yoshitsugu Kobayashi^2^, Paul J. McCarthy^3^, Tomonori Tanaka^2^, Ronald S. Tykoski^1^, Yuong-Nam Lee^4^, Ryuji Takasaki^2^, Junki Yoshida^2^

^1^ Perot Museum of Nature and Science, Dallas, Texas, U.S.A.,

^2^ Hokkaido University Museum, Hokkaido University, Hokkaido, Japan,

^3^ University of Alaska, Department of Geosciences, Fairbanks, Alaska, U.S.A.,

^4^ Seoul National University, School of Earth and Environmental Sciences, Seoul, South Korea

**The affinities of *Magnoavipes***

There has been some controversy regarding the ichnogenus *Magnoavipes*. The discussion centers around whether the maker of this ichnogenus was a large bird as first suggested [1], or a non-avian theropod [2,3], an issue of broader concern than just this ichnogenus due to the similarity in morphology and size between some non-avian and avian theropod tracks. Some recent discussion and works seem to have obfuscated the original determination of this ichnogenus as an avian theropod as put forth by Lee [1]. In the wonderfully titled book, **Tracking & the Art of Seeing**, Rezendes [4] provided a rationale for understanding the nature of tracks and what they tell about the animals being studied. In order to draw conclusions about fossil tracks, the use of modern tracks is of fundamental importance in interpreting the nature of the fossil trackmaker. In other words, it is the vehicle by which to connect to the long dead trackmaker. Therefore, by using a robust dataset of extant bird track data, gathered from published illustrations and photographs, we hope to clarify the controversy surrounding the ichnogenus *Magnoavipes*.

**Previous parameters for fossil avian tracks**

In one of the first attempts to provide criteria for the identification of fossil avialan tracks, Lockley et al. [5] suggested a list of parameters. Those criteria included that the tracks have: a) small size; b) indistinct toe pad impressions; c) a posteriorly directed hallux impression; d) slender claw impressions; e) curvature of the claw impressions of digits II and IV away from digit III; and g) slender digit impressions. Lockley et al. [5] further emphasized that there are wide divarication angles between digits II and IV (110–120^o^ or more) in fossil bird tracks. Another criterion put forth by Lockley et al. [5] was that the tracks should have a similar appearance to modern birds, a perspective that is decidedly qualitative rather than quantitative. De Valais and Melchor [6] reviewed these and additional characters for the identification of bird tracks in the fossil record. Of relevance to this discussion is the latter authors’ reiteration of the point made by Lockley et al. [5], and these authors put emphasis on qualitative characters such as similarity to modern bird tracks, and slender digit impressions.

**Arguments against an avian origin for *Magnoavipes***

Lockley et al [2] described a number of tracks generally similar in form and age to *Magnoavipes* *lowei* from Colorado and New Mexico, assigned them to a new ichnospecies *M. caneeri*, but argued that *Magnoavipes* tracks were more likely made by a non-avian theropod. They postulated these slender-toed tracks were most likely made by ornithomimid theropods, and not by large avian taxa. One of the justifications for that determination was criticism of the method of tracing digit axes to find divarication angles by Lee [1] in his description of *M. lowei* tracks. Lockley et al., [2] charged that Lee [1] had erroneously derived the wide, avian-like derivation angles of *M. lowei* tracks “by drawing a line from the tip of each lateral toe to the point at which the central axis of digit III intersected the rear of the footprint”, and that instead “Most paleoichnologists measure the angles at the intersection points of lines drawn down the axis of each digit” [7]. From their estimation, the divarication angle of figured *M. lowei* tracks were only 83° and 80° respectively, far less than the 109° to 118° determined by Lee [1] and well within the range of their newly erected *M. caneeri* [2]. They also attacked the statement by Lee [1] that *M. lowei* could have been made by a heron-like bird, a claim made based upon the general similarities of the extremely narrow *M. lowei* tracks to the exceptionally slender toes found in modern herons. Lockley et al [2] took great lengths to demonstrate *M. lowei* tracks differed in ways from modern heron tracks, offering that as additional evidence against the avian nature of the fossil track maker. The exceptional narrowness of digit impressions in *M. lowei* were also taken to task, with the assertion made that this was an artifact of the substrate having been especially soft at the time the tracks were made, resulting in collapse of the digit impressions as the trackmaker withdrew the foot [2]. At the well-defined ichnofossil level containing the original *Magnoavipes* tracks, there is no evidence to support that the tracks are undertracks. Rather they, and the other tracks found at this site are true natural impressions on a mud film overlying sand. This is further supported by the large ornithopod tracks on the horizon showing pushed-up sediment at the metatarsal-phalangeal pad, (i.e. displacement rims [8]), a key characteristic for identifying surface tracks. Lastly, mud film is known as playing a significant role in preserving the detailed morphology of tracks [9].

In a more recent effort to address the non-avian versus avian status of the ichnotaxon *Wupus agilis*, Xing et al. [3] re-examined some of the earlier metrics used to discern fossil bird tracks versus similary-sized non-avian theropod tracks. They followed Lockley et al [2] and assumed that all *Magnoavipes* isp. tracks were made by a non-avian, probably ornithomimid-like theropod. Xing et al. [3] found that a number of determinants used by previous workers were unreliable for separating avian tracks from non-avian theropod tracks. One example given by Xing et al. [3], was the divarication angle between digits II and IV overlapped in many of the taxa they sampled, with many bird tracks bearing angles less than 90° (the maximum cut-off for non-avian theropods) and some assumed non-avian ichnotaxa with angles above 90° (including their large sample of *Magnoavipes* tracks, mean angle of 93.4°) or even greater than 100° (the proposed minimum angle of avian tracks). The angle for *Wupus agilis*, the ichnotaxon of interest in their study which they concluded represented avian tracks, was calculated to have a mean of 97.5°.

Of note, figure 3 of Xing et al. [3] clearly illustrated the system of landmarks and angles used to make measurements in their study. Close examination of the figure shows the authors calculated the divarication angle between digits II and IV by drawing digit axes from the distal tip of each digit to a point near the rear edge of the print aligned with the axis of digit III. In doing so they clearly departed from the main axes of digit II and IV to make the necessary intersection. This was the same methodology harshly criticized by Lockley et al [2], which they contended produced erroneously large divarication angles in *Magnoavipes lowei* [1].

Xing et al [3] concluded that divarication angle between digits II and IV, overall size of track length, and ratio of track length versus width, all of which had been used by prior workers to differentiate fossil avian and non-avian theropod tracks, were actually unreliable tools for making that determination. They did conclude that the ratio of footprint length to pace length was a good indicator of avian affinities, with higher ratios in avian ichnotaxa and modern avians (0.34 to 0.47) and low ratios in ichnotaxa predetermined to be non-avian (0.18 to 0.31). In the judgement of Xing et al [3], this indicated that wading birds, the most likely behavior engaged in by the fossil trackmakers and the extant taxa, consistently took shorter steps, or had relatively shorter legs, than non-avian theropods. This in turn was cited as the one relatively reliable metric that could be used to discern the difference in fossil tracks. Their multivariate statistical analysis also tended to produce two main clusters of similarity – one group comprised of larger tracks with smaller divarication angles and larger pace angulation and length that included their predetermined non-avian theropods, and a second group with generally smaller tracks with wider divarication angles and lower pace angulation and pace length that encompassed most of their avian track makers.

**The case for an avian *Magnoavipes***

We follow up on some of the lines of reasoning applied by Xing et al. [3] in their assessment of some individual metrics and their use in determining avian versus non-avian fossil trackmakers. In particular, we look more closely at modern taxa and real-world behaviors of those taxa. We also look more critically at the first arguments put forth against an avian trackmaker for *Magnoavipes* [2]. Lockley et al [2] described tracks from Colorado that they attributed to a new ichnospecies within *Magnoavipes*, *Magnoavipes caneeri*. They noted that there were large morphological differences between the original tracks described by Lee [1] and the ones described from Colorado [2].

**Size as a limiting factor**

One of the key points that seemed to be problematic for the authors was the size of the original tracks described by Lee [1] and what that would mean for understanding Cenomanian vertebrate faunas. Indeed, a previously published determing factor for the avian status of a fossil trackmaker was ‘small size’ [5]. A large number of field guides are now available to assist the study of modern birds [4, 10-16], and these guides identify a great variety of modern avian trackmakers and bird behaviors. S1 Fig is a graphical compilation of published bird track sizes that includes 29 modern birds (including moas), 3 sets of tracks from large Cenozoic birds, the original report of *Magnoavipes* *lowei* from the Cenomanian of Texas [1], and the additional ichnospecies *M. denaliensis* from the Campanian-Maastrichtian of Alaska [17]. It is clear from this comparison that *Magnoavipes* tracks fall well within the known size range of large but unambiguously avian feet and tracks. It is also clear that ‘small size’ [5] is not a viable means of determining the avian or non-avian affinities of a trackmaker, further supporting that conclusion by Xing et al., [3]. Lastly, the fossil bird *Gargantuavis* from France [18-21] shows that at least one ostrich-sized bird is known from the Campanian-Maastrichtian, fully de-bunking the argument that ‘small size’ is a requirement for identifying fossil bird tracks. Therefore, the growing body fossil record opens up the plausibility of the original assertion by Lee [1] that large birds were present near or at the time that the *Magnoavipes lowei* tracks were made.

**S1 Fig. Size of Magnoavipes tracks compared to unequivocal bird tracks**. Graph showing published bird track sizes (mean print length in centimeters) of 29 large modern birds, three Cenozoic birds, and the Cretaceous ichnotaxon *Magnoavipes*. Black bars indicate modern, extant species. Gray bars indicate modern but extinct moas. Three sets of tracks from large Cenozoic birds are shown by tan bars. *Magnoavipes* *lowei* from the Cenomanian of Texas, and *M. denaliensis* from the Campanian-Maastrichtian of Alaska are depicted with light green bars.

**Effect of substrate on track-making**

Martin [22] identified three major factors that contribute to the overall gestalt of traces; aspects of the substrate, the anatomy of the trackmaker, and the behavior of the trackmaker. Elaborating on this first factor, Rezendes [4] and Elbroch and Marks [15] recognized that morphological variation within modern tracks of known trackmakers, caused by variability in sediment texture, consistency, and moisture, can produce tracks that belong to more than one morphological group. In some examples, the variability in these substrate features can even alter the number of digit impressions. Fiorillo [23] illustrated an example of a modern shorebird trackway along the bank of a river that showed tracks changing from tridactyl to didactyl on the same trackway due to the variability of moisture content and grain size of the substrate along the individual’s path of travel. Extrapolating that modern example to the fossil record, an incomplete or fragmented sample of tracks from that trackway would be easily misidentified as having been generated by more than one taxon from very different theropod clades as a result of those differences in digit impression preservation.

**Anatomy matching the trackmaker**

The similarity between the tracks created by extinct, non-avian theropods and avian theropods is obvious, and in some cases there is greater similarity in track form between large, often flightless birds and extinct non-avian theropods, than there is between groups of modern birds (S2 Fig). The tracks attributed to the Recent but extinct, large-bodied and flightless moas (S2B, C Fig) more closely resemble non-avian theropods (S2A Fig) than even relatively large flying birds (S2D Fig). The broad, weight-bearing digits of large flightless birds and flightless non-avian theropods can likely be attributed to convergent function. Meanwhile, the long, very slender pedal digits of even large flying birds is notable. It is unclear whether the exceptionally narrow digits of flying birds is entirely functional is origin, with reduced locomotor and weight-bearing needs alleviating the need for powerful, thicker digits, or if it is more closely tied to phylogeny.

**S2 Fig. Outline drawings of tridactyl theropod tracks**. A, non-avian theropod footprint from Early Cretaceous Glen Rose Limestone, Texas. B and C moas [28, 29]. D, modern Sandhill Crane [22]. E, *Magnoavipes* [1]. Note greater similarity between A, B, and C than to D and E, and greater similarity between D and E than to flightless non-avian and avian theropods. All tracks displayed to same overall length.

With respect to anatomy of trackmakers, within reviews of modern bird tracks [4, 15-16] there is significant variance in morphology of tracks across the clades of birds. For example, some modern bird tracks, such as those made by the wild turkey (*Meleagris gallopavo*; [15]) and particularly the bald eagle (*Haliaeetus leucocephalus*; [15]), show very distinct toe pad impressions even though these modern species are in different avian clades and engage in very different functional actions with their feet. Thus, the presence or absence of toe pads [5] is not a valid means for identifying bird tracks in the fossil record. In addition, some bird tracks such as those of the Piping Plovers (*Charadrius melodus*) and Sanderlings (*Calidris alba*) do not show digit I impressions because that digit has been reduced or lost [15]. In many cases, extant bird tracks do not exhibit distinct claw marks [4, 15].

In the case of *Magnoavipes* isp, Lockely et al [2] suggested these tracks were laid down by ornithomimid theropods, a point later reinforced by their inclusion in a new ichnofamily, Ornithomimimipodidae [24]. A line of anatomical evidence cited by Lockley et al [24] in the diagnosis of the new ichnofamily was the separation of the proximal part of digit II from the metatarsal pad impression and digit III (a pronounced ‘theropod gap’), a track feature that would be consistent with the proximally placed distal end of metatarsal II in the foot skeleton of many ornithomimid taxa [25]. Yet, the tracks of *Magnoavipes lowei* bear a confluent digit II fully connected to the metatarsal pad impression at the base of digit III [1]. Even some of the tracks of *M. caneeri* figured by Lockley et al [2] show no gap between digit II and III, while others are merely partial toe impressions that do not preserve the metatarsal pad impression. This would seem inconsistent with the skeletal anatomy of known ornithomimid taxa. Admittedly, one must hold out the possibility that an unknown ornithomimid taxon with a distinctly non-ornithomimid-like, distally elongated metatarsal II anatomy could have been present in the Cenomanian (*M. lowei*, *M. caneeri*) of Appalachia and Laramidia, as well as in the Campanian-Maastrichtian (*M. denaliensis*) of Beringia respectively at the times *Magnoavipes* tracks were being made.

Similarly, requiring a wide (>100°) divarication angle to be considered a fossil bird track [5] is also fraught with problems. Examination of the divarication angles of some modern birds shows significant variance in divarication angles across the clade, even within a single species S3 Fig. An exceptional example of the latter is the Killdeer (*Charadrius morinellus*), for which Elbroch and Marks [15] figured a single trackway in which the divarication angle between digits II and IV ranged from 38^°^ to 131^°^. Indeed, many modern avian taxa regularly leave tracks with divarication angles well within the range of ‘non-avian’ theropod tracks (S3 Fig). These comparisons of modern taxa indicate that relying on divarication angles between digits is of extremely limited use, if of use at all, in identifying fossil tracks as avian or not, again supporting that conclusion by Xing et al [3].

**S3 Fig. Range of avian pedal digit II-IV divarication angles**. Graph shows pedal digit II-IV divarication angles (in degrees) of 35 modern birds based on published images, shown with solid black bars. Divarication angles for *Magnoavipes lowei* and *M. denaliensis* are shown by light green bars. Length of thick bars indicates the range of measured angles for the tracks of each taxon. Lower, white-background part of graph indicates the range of divarication angles for tracks typically considered to be made by ‘non-avian theropods’. Pink background indicates part of graph with divarication angles typically accepted as being made by avian theropods (birds).

Lastly, the most heavily emphasized character in the original description of *M. lowei* tracks was the long slender digits [1], a point of significance in the determination of *M. denaliensis* as well [17]. We agree with all previous contributing authors to this discussion that determining fossil avian tracks from non-avian theropod tracks is challenging. We submit, however, that tracks that express long slender digits can be reasonably attributed to fossil birds, and further the trackmakers for this style of track were likely capable of flight. In tracks where the digits are more robust, the issue becomes murkier given the similarity between tracks made by flightless birds such as emus and moas, and non-avian theropods [26, 27]. These reviews confirm the proposal put forth by some workers studying the fossil record that no single attribute of morphology is reliable [3, 6].

**Behavior influences trackmaking**

With respect to Martin’s [22] criterion regarding behavior, casual observation of animals would reveal that animals change pace for a variety of reasons depending on behaviors such as travel (moderate to long pace length), pursuit or fleeing (very long pace length), feeding (very short pace length), etc. That high degree of behavioral variability is acknowledged by Xing et al., [3], but they still insist on pace length as one of the most reliable metrics for identifying avian tracks in their study. However, unless there is a plethora of trackways of a given ichnogenus available for study, in the absence of evidence for a particular mode of behavior when the tracks were made it seems that the use of pace length should be viewed as suspect. To further illustrate the limited use of a footprint length (FL) to pace length (PL) ratio in identifying taxa in the fossil record, S4 Fig shows modern bird tracks left in concrete on a sidewalk in Dallas, Texas, as well as two additional published trackways [28, 29]. With respect to the tracks preserved in the concrete sidewalk in Dallas (S4 FigA), the size and shape of the tracks matches well with figured tracks of grackles (*Quiscalus*; [15]). The tracks preserved in concrete can be attributed to a member of this genus, and given the abundance of the Common Grackle (*Quiscalus quiscula*) in Dallas, it is likely that this is the specific trackmaker. The average footprint length of the tracks preserved in the sidewalk is 5.2 cm, and the pace length is 23.6 cm, yielding a FL/PL of 0.22. Two additional values are provided in S4 Fig from published photographs of modern birds (S4 Fig B, C). The three values calculated from these examples of modern bird trackways are well below even the ratios identified by Xing et al. as attributable to non-avian theropods. The ratio of FL/PL is 0.18 in *Magnoavipes lowei*. Also, as an aside, note that divarication angle between digits II and IV of a common grackle and a spotted thick-knees is less than 90^°^, further invalidating the tracks of these two taxa for consideration as avian tracks based on some previous works [5].

**S4 Fig. Three examples of modern bird trackways**. **A**, tracks of a Common Grackle (ratio of FL/PL = 0.24); **B**, tracks of a Spotted Thick-knees (ratio of FL/PL = 0.19) Reprinted from [26] under a CC BY license, with permission from [Chris & Mathilde Stuart], original copyright [2013].; **C**, tracks of a Ring- necked Pheasant (ratio of FL/PL=0.16) Reprinted from [27] under a CC BY license, with permission from [Donald McLeod], original copyright [2012] . FL = Foot Length. PL = Pace Length.

Given the three major factors as put forth by Martin [22], when it comes to identifying fossil footprints, though an admittedly qualitative measure, the overall similarity to modern bird tracks remains an appropriate basis for determining the tracks of non-avialian theropods in the fossil record. Based on the points raised here, the conclusion of Lockley et al [2] that *Magnoavipes* represents tracks of ornithomimid theropods can no longer be supported by the evidence. In turn, the original interpretation of *Magnoavipes* *lowei* tracks being avian in origin [1] cannot be ruled out. It may also be that the morphological differences between *Magnoavipes lowei* and *M. caneeri* pointed out by Lockley et al. [2], are evidence that the ichnotaxon *M. caneeri* may be from a non-avian taxon, while the evidence for an avian trackmaker being responsible for the original *M. lowei* of Lee [1] remains valid. Given that, we consider the *Magnoavipes*-like tracks found in ANIA and described in the main body of text as having been made by an avian taxon..

**References**

1. Lee, YN. 1997. Bird and dinosaur footprints in the Woodbine Formation (Cenomanian), Texas. Cretaceous Research 1997; 18:849-864.
2. Lockley M, Wright, J, Matsukawa, M. A new look at *Magnoavipes* and so-called “Big Bird” tracks from Dinosaur Ridge (Cretaceous, Colorado). The Mountain Geologist 2001; 38:137-146.
3. Xing, L, Buckley, LG, McCrea, RT, Lockley, MG, Zhang, J, Piñuela, L, Klein, H, Wang, F. Reanalysis of *Wupus agilis* (Early Cretaceous) of Chongqing, China as a large avian trace: differentiating between large bird and small non-avian theropod tracks. PLoS ONE 2015; 10(5): e0123039, doi: 10.1371/journal.pone.0124039.
4. Rezendes, P. Tracking and the Art of Seeing: how to read animal tracks and sign. 2nd edition. New York: Harper Collins Publishers; 1999.
5. Lockley, MG, Yang, SY, Matsukawa, M, Fleming, F, Lim, SK. The track record of Mesozoic birds: evidence and implications. Philosophical Transactions of the Royal Society of London 1992; Series B: Biological Sciences, 336:113-134.
6. De Valais, S, Melchor, RN. Ichnotaxonomy of bird-like footprints: an example from the Late Triassic-Early Jurassic of northwest Argentina. Journal of Vertebrate Paleontology 2008; 28:145–159.
7. Leonardi, G. Glossary and manual of tetrapod footprint palaeoichnology. Brazil: Department Nacional de Producao Mineral; 1987.
8. Manning, PL. A new approach to the analysis and interpretation of tracks: examples from the Dinosauria. In McIlroy D, editor. The Application of Ichnology to Palaeoenvironmental and Stratigraphic Analysis. Geological Society of London Special Publication 228. London; 2004. p. 93–123.
9. Paik, IS, Kim, HJ, Lee, H, Kim, S. A large and distinct skin impression on the cast of a sauropod dinosaur footprint from Early Cretaceous floodplain deposits, Korea. Scientific Reports 2017; 7:16339.
10. Murie, OJ. A Field Guide to Animal Tracks. Boston: Houghton Mifflin Co.; 1954.
11. Halfpenny, JC. A field guide to mammal tracking in North America. Boulder: Johnson Books; 1986.
12. Halfpenny, JC. Scats and tracks of the Pacific Coast, including British Columbia. Helena: Falcon Publishing; 1999.
13. Halfpenny, JC. Scats and tracks of Alaska, including the Yukon and British Columbia. Helena: Falcon Publishing, Inc.; 2007.
14. Forrest, LR. Field Guide to Tracking Animals in Snow. Mechanicsburg: Stackpole Books; 1998.
15. Elbroch, M, Marks, E. Bird Tracks and Sign. Mechanicsburg: Stackpole Books, Mechanicsburg: 2001.
16. Brown, R, Ferguson, J, Lawrence, M, Lee, D. Tracks & signs of the birds of Britain & Europe. 2nd edition. London: Christopher Helm; 2003.
17. Fiorillo, AR, Hasiotis, ST, Kobayashi, Y, Breithaupt, BH, and McCarthy, PJ. Bird tracks from the Upper Cretaceous Cantwell Formation of Denali National Park, Alaska, USA: a new perspective on ancient northern polar vertebrate biodiversity. Journal of Systematic Palaeontology 2011; 9:33-49.
18. Buffetaut,E, Le Loeuff, J, Mechin,P, Mechin-Salessy, A. A large French Cretaceous bird. Nature 1995; 377:110
19. Buffetaut, E, Le Loeuff, J. A new giant ground bird from the Upper Cretaceous of southern France. Journal of the Geological Society 1998; 155:1-4.
20. Buffetaut, E, Angst, D, Mechin, P, Mechin-Salessy, A. New remains of the giant bird *Gargantuavis philoinos* from the Late Cretaceous of Provence (south-eastern France). Palaeovertebrata 2015; 39:1-6.
21. Buffetaut, E, Angst, D. A femur of the Late Cretaceous giant bird Gargantuavis from Cruzy (southern France) and its systematic inplications. Palaeovertebrata 2019; doi:10.18563/pv.42.1.e3.
22. Martin, AJ. Life traces of the Georgia coast: Revealing the unseen lives of plants and animals. Bloomington: Indiana University Press; 2013.
23. Fiorillo, AR. Alaska Dinosaurs: an Ancient Arctic World. Boca Raton: CRC Press; 2018.
24. Lockley, M. Cart, K, Martin, J, Milner, A. New theropod tracksites from the Upper Cretaceous “Mesaverde” Group, western Colorado: implications for ornithomimosaur track morphology. In Sullivan, RM, S. G. Lucas, SG, Spielman, JA editors. Fossil Record 3. New Mexico Museum of Natural History and Science Bulletin 53. Albuquerque, New Mexico 2011:321-329.
25. Makovicky, P, Kobayashi Y, Currie PJ. 2004. Ornithomimosauria. In Weishampel, DB, Dodson, P and Osmólska, H editors. The Dinosauria, 2^nd^ edition. Berkeley: University of California Press; p. 137-164.
26. Owen, R. Memoirs on the extinct wingless birds of New Zealand, with an appendix on those of England, Australia, Newfoundland, Mauritius, and Rodriguez. London: John Van Voorst; 1879.
27. Hill, H. 1895. On the occurrence of Moa-footprints in the bed of the Manawatu River, near Palmerston North. Transactions and proceedings of the New Zealand Institute 1895; 27:476-477.
28. Stuart, C, Stuart, T. Field guide to the larger mammals of Africa. Cape Town: Struik Publishers; 2006.
29. Don McLeod’s Nature Blog, <https://www.donaldmcleod.com/Keyword-Galleries/Bird-Tracks/Ringnecked-Pheasant/>
